# Dual Suppression of Oxidative Stress and JAK/STAT3 Signaling Attenuates Inflammation‐Driven Corneal Neovascularization

**DOI:** 10.1002/advs.78647

**Published:** 2026-11-10

**Authors:** Qinghua Li, Ruoxi Li, Bingxin Ren, Chaoya Gong, Hui Yan, Jie Chen, Benzhao Fu, Chaoqun Wei, Qingdong Bao, Yang Li, Xiaoxue Liu, Wenlong Li, Hua Gao

**Affiliations:** ^1^ State Key Laboratory Cultivation Base Shandong Provincial Key Laboratory of Ophthalmology Eye Institute of Shandong First Medical University Qingdao China; ^2^ Eye Hospital of Shandong First Medical University (Shandong Eye Hospital) Jinan China; ^3^ School of Ophthalmology Shandong First Medical University Jinan China; ^4^ State Key Laboratory of Structural Chemistry Fujian Institute of Research on the Structure of Matter Chinese Academy of Sciences Fuzhou China

**Keywords:** biomimetic nanozyme, corneal neovascularization, macrophage membrane, oxidative stress, STAT3 signaling

## Abstract

Corneal neovascularization (CoNV) is a vision‐threatening disorder driven by persistent oxidative stress and inflammation, yet effective therapies targeting these interconnected pathological processes remain limited. Here, we identify enhanced signal transducer and activator of transcription 3 (STAT3) phosphorylation and inflammatory signaling in corneal samples from patients with CoNV, as well as reactive oxygen species (ROS) accumulation and macrophage infiltration were observed in an alkali burn‐induced mouse model. To address these pathological features, we developed Sta@MSR, a macrophage membrane‐cloaked biomimetic nanozyme integrating ruthenium‐mediated ROS scavenging with Stattic‐mediated STAT3 inhibition. Mechanistically, Sta@MSR attenuated oxidative stress and STAT3‐associated inflammatory signaling, thereby suppressing endothelial angiogenic activity and pathological neovascularization. Furthermore, macrophage membrane cloaking enhanced ocular retention and corneal penetration, facilitating topical delivery. Importantly, Sta@MSR markedly reduced pathological neovascularization and inflammatory injury, restored corneal transparency, and exhibited favorable ocular and systemic biocompatibility. Collectively, this work highlights coordinated regulation of oxidative stress and STAT3 signaling as a promising therapeutic strategy for inflammation‐driven CoNV.

## Graphical abstract

Corneal neovascularization is sustained by oxidative stress and inflammatory STAT3 signaling. A macrophage membrane‐cloaked nanozyme, Sta@MSR, combines ruthenium‐mediated ROS scavenging with Stattic‐mediated STAT3 inhibition. Delivered as eye drops, Sta@MSR enhances ocular retention and corneal penetration, reduces inflammatory and angiogenic responses, and restores corneal transparency, offering a coordinated topical strategy for treating inflammation‐driven corneal neovascularization.

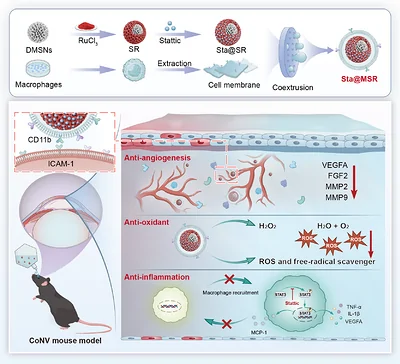

## 1 Introduction

Corneal neovascularization (CoNV) is a major cause of corneal blindness and vision impairment, as pathological vessel invasion disrupts the physiological avascular and highly organized stromal microenvironment required for corneal transparency [1, 2, 3]. Newly formed vessels facilitate inflammatory cell infiltration and extracellular matrix remodeling, resulting in corneal edema, opacity, fibrosis, and irreversible visual dysfunction [4]. Alkali burns represent one of the most severe causes of CoNV because rapid chemical penetration causes extensive epithelial disruption, stromal injury, and persistent inflammatory activation [5, 6]. CoNV presents a significant global burden, with approximately 1.4 million new cases annually, yet treatment options are inadequate [7]. Long‐term corticosteroid use may cause elevated intraocular pressure, delayed epithelial healing, and infection risk, while corneal transplantation is hampered by factors including donor scarcity, high financial burden, and immune rejection [8, 9, 10]. Although anti‐vascular endothelial growth factor (VEGF) therapy is the first‐line treatment for retinal neovascular disorders, its therapeutic benefit on the ocular surface remains limited, with inconsistent outcomes in clinical practice [11, 12]. Therefore, there is an urgent need to develop effective therapeutic strategies based on the pathogenesis of CoNV.

Increasing evidence suggests that alkali burn‐induced CoNV is not a single‐factor angiogenic disease, but a complex pathological process driven by the interaction of oxidative stress, inflammation, macrophage recruitment, and angiogenic signaling [13, 14, 15]. After alkali injury, excessive reactive oxygen species (ROS) accumulate in corneal tissues and directly damage epithelial and stromal cells [16]. Meanwhile, ROS act as upstream amplifiers of inflammatory signaling, promoting the production of pro‐inflammatory cytokines and chemokines [17]. These mediators recruit macrophages and other immune cells into the injured cornea, further amplifying local inflammation and promoting VEGF‐mediated neovascularization [18]. Among the signaling pathways linking inflammation with angiogenesis, Janus kinase‐signal transducer and activator of transcription 3 (JAK‐STAT3) signaling plays a central role [19, 20]. Activated STAT3 can enhance the expression of VEGFA and inflammatory mediators, thereby sustaining the inflammatory‐angiogenic loop that drives CoNV progression [21]. Thus, simultaneous regulation of redox imbalance and STAT3‐mediated inflammatory angiogenesis may represent a more effective therapeutic strategy for CoNV.

Nanomedicine provides a promising approach for coordinated modulation of multiple pathological processes within a single therapeutic system. Nanozymes, owing to their enzyme‐mimicking catalytic activity, offer sustained and recyclable regulation of ROS compared with conventional antioxidant molecules that are rapidly consumed [22, 23]. In diverse biomedical applications, including oncology, gastrointestinal diseases, and retinal disorders, nanozymes have demonstrated potent antioxidative capacity [24, 25, 26, 27, 28, 29]. For example, Xue et al. developed a ruthenium (Ru)‐based nanozyme that effectively restored redox homeostasis and alleviated retinal neovascular pathology [30]. Although catalytic ROS scavenging can alleviate oxidative injury, antioxidant treatment alone may not fully suppress persistent STAT3‐associated inflammatory and angiogenic signaling. Conversely, pharmacological STAT3 inhibition does not directly eliminate the excessive ROS generated by chemical injury. These complementary limitations highlight the therapeutic rationale for integrating redox regulation and STAT3 inhibition within a single platform. However, successful topical ocular delivery remains challenging because therapeutic nanoparticles must overcome rapid tear clearance, blinking‐induced elimination, limited epithelial permeability, and poor retention within inflamed corneal tissues.

Biomimetic membrane cloaking has emerged as an effective strategy to improve nanoparticle delivery by transferring functional biological interfaces from parental cells [31, 32]. Macrophage membrane‐coated nanoparticles retain membrane‐associated adhesion molecules and inflammatory receptors, enabling enhanced interaction with inflamed tissues and modulation of inflammatory microenvironments [32, 33, 34, 35, 36]. Given that macrophage infiltration and inflammatory signaling are prominent features of alkali burn‐induced CoNV, macrophage membrane‐coated nanotherapeutics may provide a unique opportunity to integrate lesion‐responsive delivery with microenvironmental regulation. Nevertheless, their application as topical ocular therapeutics for simultaneously controlling oxidative stress, inflammation, and angiogenesis in CoNV remains largely unexplored.

In this study, we investigated the association between oxidative stress, inflammatory activation, and STAT3 signaling in human CoNV specimens and a mouse alkali burn model. Enhanced STAT3 phosphorylation in human CoNV corneas, together with ROS accumulation, macrophage infiltration, and STAT3 activation in injured mouse corneas, supported the concurrent involvement of these pathological processes in CoNV progression. Guided by these findings, we developed Sta@MSR, a macrophage membrane‐cloaked biomimetic nanozyme integrating Ru‐mediated catalytic ROS scavenging with Stattic‐mediated STAT3 inhibition. This integrated design combines sustained ROS scavenging with suppression of STAT3‐associated inflammatory and angiogenic signaling, while macrophage membrane cloaking facilitates interaction with the injured corneal microenvironment and improves local delivery. As an eye‐drop formulation, Sta@MSR simultaneously suppressed oxidative stress and STAT3‐associated inflammatory angiogenesis, effectively remodeling the pathological microenvironment and alleviating CoNV progression. Our findings demonstrate the therapeutic advantages of coordinating redox regulation, STAT3 inhibition, and biomimetic ocular delivery, providing a complementary approach to conventional single‐pathway interventions.

## 2 Results

### 2.1 CoNV is Characterized by Oxidative Stress, Inflammatory Activation, and JAK‐STAT3 Signaling Activation

Corneal neovascularization (CoNV) after alkali burn is not merely a consequence of excessive angiogenic stimulation, but rather a complex pathological process driven by oxidative stress, inflammatory amplification, immune‐cell infiltration, and pro‐angiogenic signaling. Defining these interconnected pathological features is essential for rationally designing a therapeutic platform capable of intervening at multiple levels of disease progression. We therefore first examined the molecular alterations associated with CoNV in human corneal tissues and further validated these pathological signatures in a mouse alkali burn‐induced CoNV model.

Human corneal tissues from normal and CoNV‐affected eyes were subjected to protein extraction and western blot analysis to evaluate angiogenic, inflammatory, and STAT3‐related signaling changes (Figure 1a). Compared with normal corneas, CoNV tissues showed markedly increased VEGFA expression, confirming the presence of a strong pro‐angiogenic stimulus in diseased corneas (Figure 1b,c). Meanwhile, representative inflammatory mediators, including inducible nitric oxide synthase (iNOS), tumor necrosis factor‐alpha (TNF‐α), interleukin‐1beta (IL‐1β), and interleukin‐6 (IL‐6), were significantly upregulated in CoNV tissues, indicating that corneal angiogenesis develops within a highly inflammatory microenvironment rather than as an isolated vascular event. Notably, phosphorylation of STAT3 (p‐STAT3) was also substantially elevated, whereas total STAT3 showed no comparable increase, suggesting that STAT3 activation, rather than simple STAT3 abundance, is associated with the pathological state of CoNV. These findings identify inflammation‐associated STAT3 activation as a potential molecular bridge linking inflammatory injury to angiogenic progression.

**FIGURE 1:**
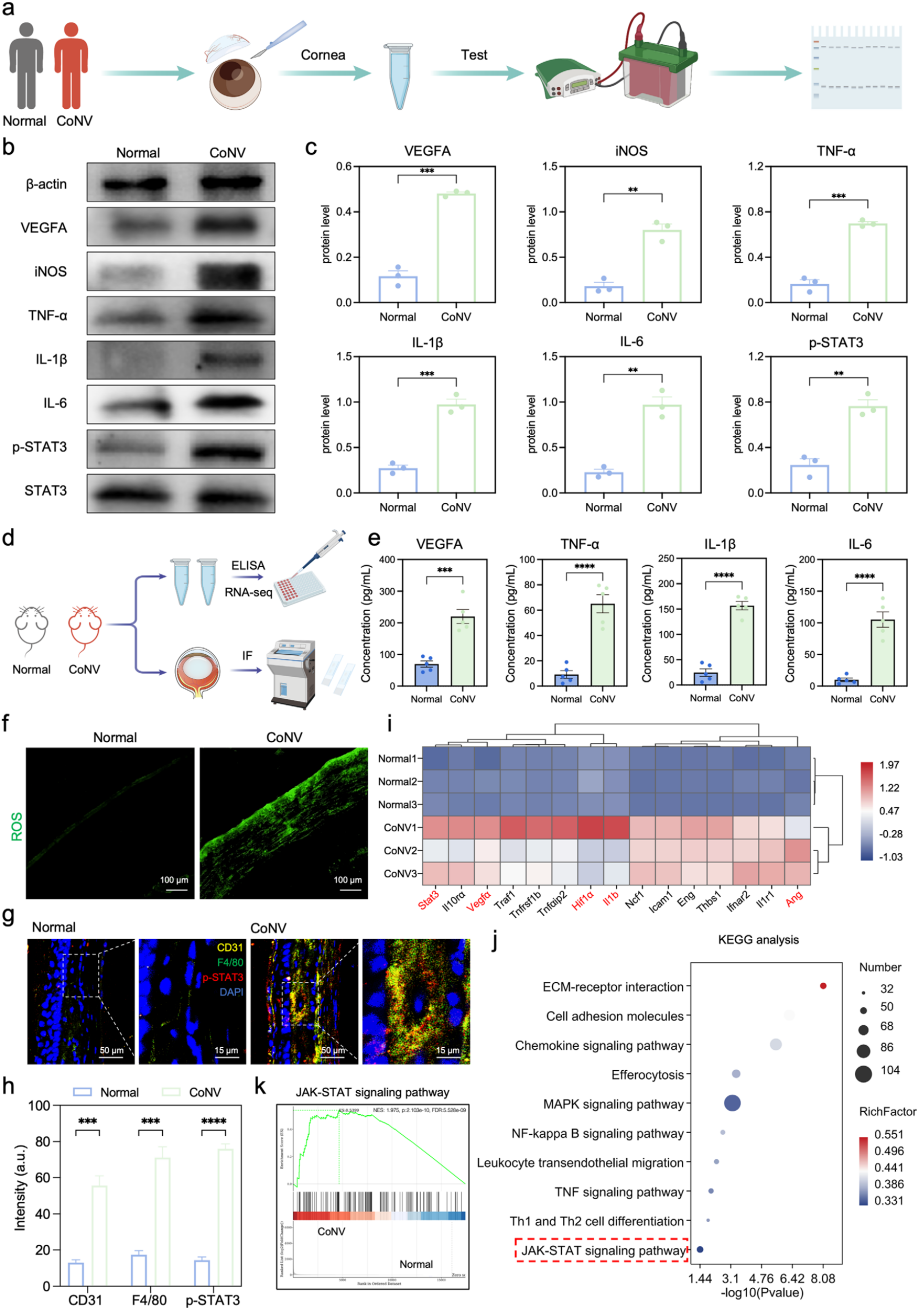
Oxidative stress, inflammatory activation, and JAK‐STAT3 activation in CoNV. (a) Schematic illustration of corneal protein extraction and western blot analysis of normal and CoNV groups. (b) Western blot analysis of VEGFA, iNOS, TNF‐α, IL‐1β, IL‐6, p‐STAT3, and STAT3 expression in corneal tissues. (c) Quantitative analysis of VEGFA, iNOS, TNF‐α, IL‐1β, and IL‐6 protein expression, together with the p‐STAT3/total STAT3 ratio used to assess STAT3 phosphorylation (*n* = 3). (d) Schematic of corneal tissue collection from normal and alkali burn‐induced CoNV mice for subsequent assays. (e) ELISA quantification of VEGFA, TNF‐α, IL‐1β, and IL‐6 levels in normal and CoNV mouse corneas (*n* = 5). (f) Representative CellROX fluorescence images showing oxidative stress‐associated fluorescence in normal and CoNV mouse corneas. Scale bars, 100 µm. (g) Representative immunofluorescence images of CD31, F4/80, and p‐STAT3 expression in normal and CoNV mouse corneas. Nuclei were counterstained with DAPI. Scale bars, 50 µm and 15 µm. (h) Quantitative fluorescence intensity analysis of CD31, F4/80, and p‐STAT3 in cornea (*n* = 3). (i) Heatmap showing differentially expressed angiogenesis, inflammation, and STAT3‐related genes in normal and CoNV mouse corneas. (j) KEGG pathway enrichment analysis of differentially expressed genes in normal and CoNV mouse corneas. (k) GSEA showing enrichment of the JAK‐STAT signaling pathway in CoNV mouse corneas. Data are presented as mean ± SEM. Data in (c,e,h) analyzed by two‐tailed Student's t‐test. ^**^ *p* < 0.01, ^***^ *p* < 0.001, ^****^ *p* < 0.0001.

To determine whether these pathological characteristics could be recapitulated in vivo, we established a mouse model of alkali burn‐induced CoNV and collected corneal tissues for cytokine analysis, ROS detection, immunofluorescence staining, and transcriptomic profiling (Figure 1d). Consistent with the observations in human corneal tissues, enzyme‐linked immunosorbent assay (ELISA) analysis showed significantly elevated levels of VEGFA, TNF‐α, IL‐1β, and IL‐6 in alkali‐burned mouse corneas compared with normal controls (Figure 1e). These results confirm that alkali injury rapidly establishes a pro‐inflammatory and pro‐angiogenic corneal microenvironment. In parallel, CellROX fluorescence staining revealed markedly increased oxidative stress‐associated fluorescence in CoNV corneas compared with normal corneas (Figure 1f). This pronounced oxidative stress is highly relevant to disease progression, as excessive ROS can act not only as direct cytotoxic mediators but also as upstream amplifiers of inflammatory and angiogenic signaling.

Immunofluorescence staining further demonstrated extensive CD31‐positive neovascularization in alkali‐burned corneas, accompanied by abundant F4/80‐positive macrophage infiltration and strong p‐STAT3 fluorescence signals within the injured region (Figure 1g,h). The spatial coexistence of vascular ingrowth, macrophage accumulation, and STAT3 activation suggests that CoNV progression involves coordinated crosstalk between infiltrating immune cells and angiogenic signaling pathways. This is particularly important because macrophage‐derived cytokines can sustain inflammatory activation, while STAT3 signaling can further promote VEGFA expression and vascular remodeling, thereby forming a self‐reinforcing inflammatory‐angiogenic loop.

To further characterize the molecular network underlying CoNV progression, RNA sequencing (RNA‐seq) was performed on normal and CoNV mouse corneas. Heatmap analysis revealed prominent upregulation of angiogenesis‐, inflammation‐ and STAT3‐related genes, including *Stat3*, *Vegfa*, *Hif1a*, *Il1b* and *Ang*, in CoNV tissues (Figure 1i). Kyoto encyclopedia of genes and genomes (KEGG) enrichment analysis showed that differentially expressed genes were enriched in multiple immune‐ and inflammation‐associated pathways, including TNF signaling, NF‐κB signaling, chemokine signaling, and leukocyte transendothelial migration (Figure 1j). Importantly, the JAK‐STAT signaling pathway was also enriched, and gene set enrichment analysis (GSEA) further showed positive enrichment of JAK‐STAT signaling in CoNV tissues (Figure 1k). These transcriptomic data support the biochemical and histological findings, indicating that STAT3‐associated signaling is deeply involved in the inflammatory and angiogenic remodeling of alkali‐burned corneas.

Collectively, these results demonstrate that alkali burn‐induced CoNV is governed by an oxidative‐inflammatory‐angiogenic cascade characterized by ROS accumulation, inflammatory cytokine release, macrophage infiltration, JAK‐STAT3 pathway activation, and VEGFA‐driven neovascularization. This pathological landscape provides a clear therapeutic rationale for developing a biomimetic nanoplatform that simultaneously scavenges ROS, intercepts inflammation, and suppresses STAT3‐mediated angiogenic signaling, rather than targeting angiogenesis alone.

### 2.2 Preparation and Characterization of Sta@MSR

Guided by the oxidative‐inflammatory pathological features identified in CoNV, we next engineered a biomimetic nanoplatform capable of simultaneously delivering a STAT3 inhibitor, scavenging ROS, and interfacing with the inflammatory microenvironment. As illustrated in Figure 2a and Figure S1, dendritic mesoporous silica nanoparticles (DMSNs) were first synthesized as a high‐surface‐area scaffold, followed by Ru modification to generate Silica‐Ru nanoparticles (SR), thereby endowing the framework with antioxidative activity. Taking advantage of the porous architecture of SR, Stattic was incorporated into SR to obtain Stattic‐loaded SR nanoparticles (Sta@SR) (Figure 2b). High‐performance liquid chromatography (HPLC) was performed to evaluate the encapsulation efficiency (EE) and loading content (LC) of Stattic in Sta@SR. Stattic was efficiently entrapped within SR, with an EE of 77% and LC of 8.5%, confirming the favorable drug‐loading capacity of the SR scaffold. The resulting Sta@SR nanoparticles were then cloaked with macrophage membrane to obtain the biomimetic formulation Sta@MSR (Figure 2b). This hierarchical design integrates structural support, catalytic antioxidation, molecular inhibition of STAT3 signaling, and macrophage membrane‐mediated inflammatory interfacing within a single nanosystem.

**FIGURE 2:**
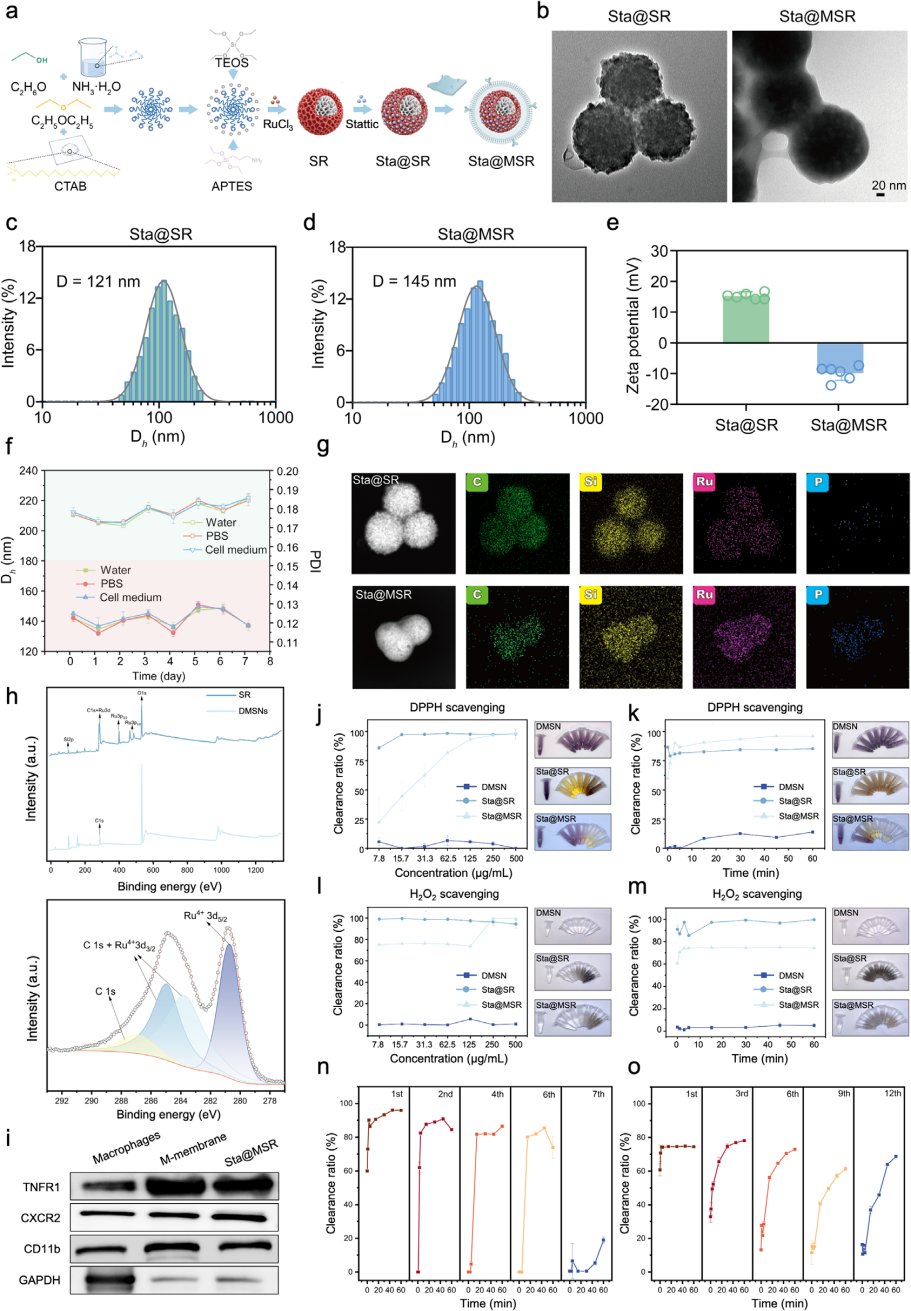
Construction and characterization of the Sta@MSR nanoplatform. (a) Schematic illustration of the synthesis of Sta@MSR. (b) Representative TEM images of Sta@SR and Sta@MSR. (c) Hydrodynamic size distributions of Sta@SR. (d) Hydrodynamic size distributions of Sta@MSR. (e) Zeta potentials of Sta@SR and Sta@MSR (*n* = 6). (f) Colloidal stability of Sta@MSR in water, PBS, and cell culture medium (*n* = 6). (g) Elemental mapping of Sta@SR and Sta@MSR showing C, Si, Ru, and P distribution. (h) XPS survey spectra of DMSNs and SR, together with the high‐resolution Ru 3d spectrum of SR, confirming the successful incorporation of Ru species into the silica framework. (i) Western blot analysis of macrophage membrane‐associated inflammatory receptors and adhesion proteins in macrophages, macrophage membrane, and Sta@MSR. (j,k) Concentration‐ and time‐dependent DPPH radical scavenging activity of Sta@SR and Sta@MSR (*n* = 3). (l,m) Concentration‐ and time‐dependent H_2_O_2_ scavenging activity of Sta@SR and Sta@MSR (*n* = 3). (n,o) Cyclic scavenging performance of Sta@MSR toward DPPH radicals and H_2_O_2_, respectively (*n* = 3). Data are presented as mean ± SEM.

Dynamic light scattering further showed that Sta@SR exhibited an average hydrodynamic diameter of approximately 121 nm, which increased to approximately 145 nm after membrane coating (Figure 2c,d). In parallel, the zeta potential shifted from a positive value for Sta@SR to a distinctly negative value for Sta@MSR (Figure 2e), further supporting successful surface reconstitution with macrophage membrane components. The colloidal stability of the nanoparticles was then evaluated in water, phosphate buffered saline (PBS), and cell culture medium. Sta@MSR displayed minimal changes in size and polydispersity over the observation period in all tested media (Figure 2f), indicating good dispersibility and physicochemical stability under physiologically relevant conditions. This stable behavior is particularly important for ophthalmic application, where prolonged suspension stability is favorable for reproducible corneal contact and therapeutic performance. To further verify the compositional features of the nanoplatform, elemental mapping analysis was performed. As shown in Figure 2g, Sta@SR displayed characteristic signals of C, Si, and Ru, whereas Sta@MSR additionally exhibited a clear P signal, consistent with the presence of membrane‐derived phospholipids on the nanoparticle surface. X‐ray photoelectron spectroscopy (XPS) analysis confirmed the successful incorporation of Ru into the silica framework (Figure 2h). Compared with DMSNs, SR exhibited clear Ru‐related signals in the survey spectrum, and high‐resolution Ru 3d analysis further verified the presence of Ru^4^^+^ species. These results demonstrate the formation of a Ru‐modified silica scaffold, providing the compositional basis for subsequent catalytic ROS‐scavenging activity.

Sodium dodecyl sulfate‐polyacrylamide gel electrophoresis (SDS‐PAGE) protein profiling showed that Sta@MSR retained a protein band pattern similar to that of isolated macrophage membranes, indicating successful inheritance of membrane‐associated proteins after cloaking (Figure S2). Western blot analysis showed that representative inflammatory recognition/adhesion‐related proteins, including tumor necrosis factor receptor 1 (TNFR1), C‐X‐C motif chemokine receptor 2 (CXCR2), and cluster of differentiation 11b (CD11b), were present in isolated macrophage membranes and Sta@MSR, whereas the cytoplasmic protein (GAPDH) was markedly reduced (Figure 2i). TNFR1 recognizes the pro‐inflammatory cytokine TNF‐α, CXCR2 participates in inflammatory chemokine sensing, and CD11b contributes to intercellular adhesion molecule‐1 (ICAM‐1)‐mediated adhesion at inflamed tissues [37, 38, 39]. The preservation of these membrane proteins suggests that the membrane shell may retain biological functions beyond passive camouflage, potentially enabling inflammatory microenvironment recognition, cytokine interception, and enhanced lesion homing.

We next assessed the antioxidant activity of the nanoplatform. The 2,2‐diphenyl‐1‐picrylhydrazyl (DPPH) assay was used to evaluate free radical‐scavenging capacity, while the H_2_O_2_ assay assessed the removal of a biologically relevant non‐radical ROS associated with oxidative stress and inflammation [40, 41]. In the DPPH assay, both Sta@SR and Sta@MSR exhibited concentration‐dependent radical scavenging activity, with high clearance efficiency achieved across increasing concentrations (Figure 2j). Consistently, ultraviolet‐visible (UV–vis) absorption spectra and electron paramagnetic resonance (EPR) analysis further showed that the characteristic DPPH signals gradually decreased with increasing Sta@MSR concentration, confirming the dose‐dependent free radical scavenging ability (Figure S3a,b). Time‐dependent measurements similarly showed rapid and sustained DPPH scavenging behavior for both formulations (Figure 2k). These results show that membrane coating did not compromise the radical scavenging capability of the Ru‐decorated core, indicating that the biomimetic shell remained sufficiently permissive to preserve catalytic antioxidant function.

Given that H_2_O_2_ is a major ROS species involved in oxidative injury and inflammatory amplification, this catalytic clearance ability is highly relevant to the pathological microenvironment of alkali‐burned corneas. A similar trend was observed in the H_2_O_2_ scavenging assay. Both Sta@SR and Sta@MSR showed effective concentration‐dependent elimination of H_2_O_2_ (Figure 2l), and time‐course measurements further confirmed robust and rapid peroxide scavenging (Figure 2m). To further evaluate the persistence of antioxidant activity, cyclic scavenging assays were performed to determine whether Sta@MSR could maintain its ROS‐scavenging capacity under repeated oxidative challenges. As shown in Figure 2n,o, Sta@MSR retained its ability to scavenge DPPH radicals and H_2_O_2_ over multiple cycles. This sustained scavenging performance suggests that Sta@MSR can participate in successive redox reactions, supporting its catalytic antioxidant characteristics and potential for prolonged regulation of oxidative stress.

Collectively, these results demonstrate that Sta@MSR was successfully constructed as a stable biomimetic nanoplatform with integrated drug loading, catalytic antioxidative, and inflammation interfacing functions. The dendritic mesoporous silica Ru scaffold supports Stattic loading and sustained ROS scavenging, while macrophage membrane cloaking preserves key proteins associated with inflammatory recognition and adhesion. This multifunctional architecture directly addresses the oxidative inflammatory microenvironment of CoNV and provides a foundation for subsequent therapeutic evaluation.

### 2.3 Sta@MSR Promotes Cellular Internalization and Suppresses Oxidative Stress and Inflammatory Activation

Efficient cellular internalization is important for the intracellular antioxidative and STAT3‐inhibitory functions of Sta@MSR. To mimic the inflammatory microenvironment of CoNV, human corneal epithelial cells (HCECs) were pre‐stimulated with lipopolysaccharide (LPS) to establish an inflammatory epithelial cell model [42]. We then examined the cellular internalization of Alexa Fluor 488 (AF488)‐labeled formulations in inflammatory HCECs. Confocal imaging showed weak intracellular fluorescence in the free AF488 group, while AF488@SR produced stronger signals and AF488@MSR exhibited the most pronounced intracellular fluorescence (Figure 3a). Flow cytometric analysis further confirmed this trend, showing significantly increased cellular fluorescence intensity in the AF488@MSR group compared with free AF488 and AF488@SR (Figure 3b). In addition, AF488@MSR uptake increased in a time‐ and concentration‐dependent manner, as reflected by progressively enhanced intracellular fluorescence with prolonged incubation and higher nanoparticle concentrations (Figure S4a,b). These results demonstrate that macrophage membrane cloaking facilitates cellular internalization of the nanoplatform, providing a prerequisite for subsequent intracellular bioactivity. To further investigate the influence of inflammatory stimulation on AF488@MSR uptake, we compared the cellular uptake of AF488@MSR between normal and LPS‐stimulated HCECs. As shown in Figure S5a,b, LPS stimulation significantly enhanced the cellular uptake of AF488@MSR in HCECs. These findings suggest that inflammatory conditions facilitate AF488@MSR uptake, further supporting the potential advantages of macrophage membrane cloaking for nanoparticle delivery to inflamed corneal tissues.

**FIGURE 3:**
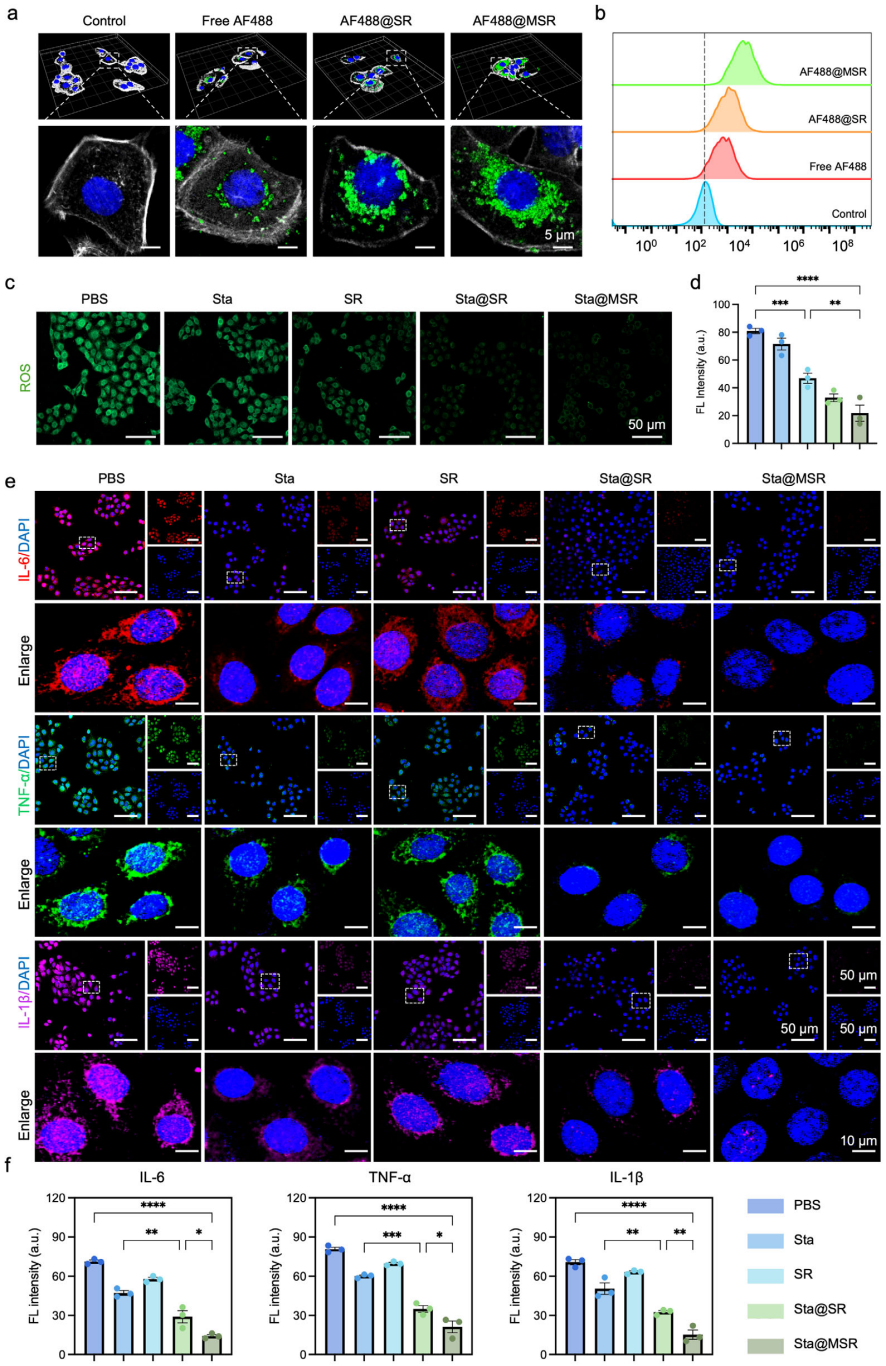
Enhanced cellular uptake and intracellular antioxidative and anti‐inflammatory activities of Sta@MSR in HCECs. (a) Representative confocal fluorescence images showing the cellular uptake in LPS‐stimulated HCECs. Nuclei were stained with DAPI. Scale bars, 5 µm. (b) Flow cytometric analysis of the cellular uptake efficiency. (c) Representative CellROX fluorescence images showing intracellular oxidative stress in H_2_O_2_‐stimulated HCECs after different treatments. Scale bars, 50 µm. (d) Quantitative analysis of CellROX fluorescence intensity in different treatment groups (*n* = 3). (e) Representative immunofluorescence images showing the expression of IL‐6, TNF‐α, and IL‐1β in LPS‐stimulated HCECs. Nuclei were stained with DAPI. Scale bars, 50 µm and 10 µm. (f) Quantitative fluorescence intensity analysis of IL‐6, TNF‐α, and IL‐1β (*n* = 3). Data are presented as mean ± SEM. Data in (d,f) analyzed by one‐way analysis of variance (ANOVA) with Tukey's multiple comparisons test. ^*^ *p* < 0.05, ^**^ *p* < 0.01, ^***^ *p* < 0.001, ^****^ *p* < 0.0001.

We next assessed whether enhanced cellular uptake translated into improved antioxidative efficacy. HCECs were stimulated with H_2_O_2_ to establish an oxidative stress model, and intracellular oxidative stress was evaluated using CellROX fluorescence staining. H_2_O_2_ stimulation induced intense fluorescence in the PBS group, indicating elevated intracellular oxidative stress (Figure 3c,d). Free Stattic (Sta) produced only a modest reduction in fluorescence intensity, whereas SR‐containing formulations markedly attenuated the fluorescence signal. Notably, Sta@MSR exhibited the lowest CellROX fluorescence intensity among the treatment groups, indicating the greatest attenuation of intracellular oxidative stress. Furthermore, concentration‐ and time‐dependent analyses revealed a progressive decrease in CellROX fluorescence following Sta@MSR treatment (Figure S6a,b). These findings support the intracellular antioxidative activity of Sta@MSR and suggest that macrophage membrane‐mediated cellular uptake may contribute to its enhanced therapeutic effects.

Because oxidative stress and inflammatory activation are closely interconnected in alkali burn‐associated CoNV, we further examined the anti‐inflammatory activity of Sta@MSR in LPS‐stimulated HCECs. Immunofluorescence staining showed markedly elevated IL‐6, TNF‐α, and IL‐1β expression in the PBS‐treated inflammatory model, confirming successful induction of a pro‐inflammatory state (Figure 3e). Free Stattic and SR each partially reduced cytokine expression, suggesting that STAT3 inhibition and ROS scavenging can individually attenuate inflammatory activation. Sta@SR produced a stronger suppressive effect than either single component, supporting the cooperative anti‐inflammatory effect of combining Stattic with the antioxidative SR scaffold. Notably, Sta@MSR most effectively reduced IL‐6, TNF‐α, and IL‐1β fluorescence signals, and quantitative analysis confirmed the superior anti‐inflammatory performance of Sta@MSR (Figure 3f).

The enhanced intracellular anti‐inflammatory efficacy of Sta@MSR likely arises from coordinated regulation of multiple pathological processes. The SR core reduces oxidative stress and thereby weakens ROS‐driven inflammatory amplification, while Stattic suppresses STAT3‐associated inflammatory signaling. Meanwhile, macrophage membrane cloaking improves cellular delivery and preserves inflammation‐associated membrane proteins, which may further contribute to inflammatory microenvironment interfacing. Collectively, these results demonstrate that Sta@MSR is efficiently internalized by HCECs and potently suppresses intracellular oxidative stress and inflammatory activation, providing a cellular mechanistic basis for its therapeutic application in the oxidative‐inflammatory microenvironment of CoNV.

### 2.4 Sta@MSR Crosses an Epithelial Barrier Model and Suppresses Endothelial Angiogenic Activity In Vitro

Given that the corneal epithelium represents a major biological barrier for topical ocular delivery, we first evaluated whether Sta@MSR could cross an epithelial barrier model. The epithelial barrier‐crossing ability of Sta@MSR was examined using a Transwell co‐culture system with an HCEC monolayer in the upper chamber (Figure 4a). Compared with free AF488, AF488@SR showed increased fluorescence in the lower chamber, while AF488@MSR produced the strongest fluorescence signal (Figure 4b). These results indicate that macrophage membrane cloaking enhances the ability of the nanoplatform to traverse the epithelial barrier model.

**FIGURE 4:**
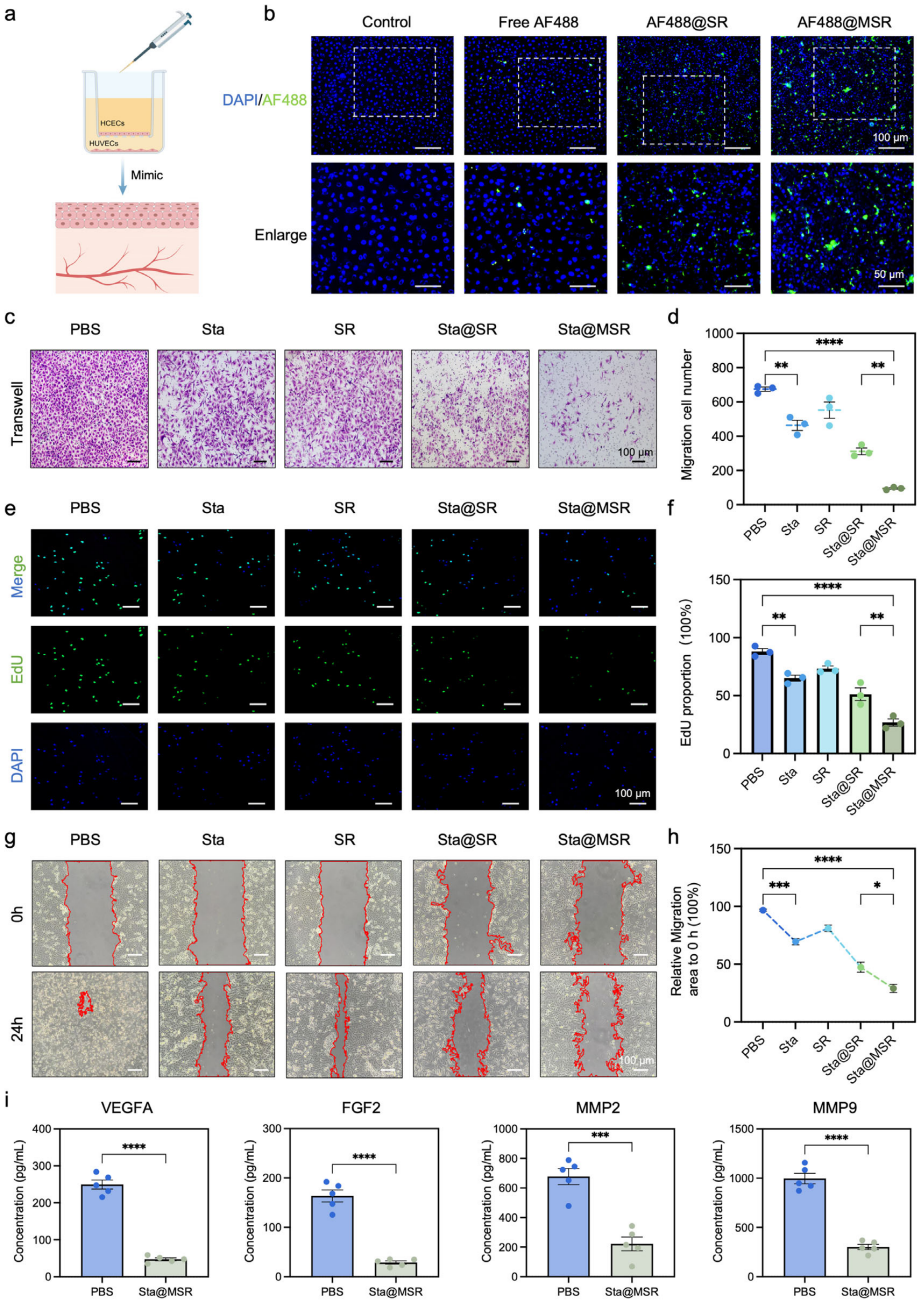
Sta@MSR crosses an epithelial barrier model and suppresses inflammation‐driven endothelial angiogenic activity. (a) Schematic illustration of the transwell co‐culture system used to mimic the corneal epithelial barrier. (b) Representative fluorescence images of the lower chamber. Nuclei were stained with DAPI. Scale bars, 100 µm and 50 µm for enlarged images. (c) Representative images of Transwell migration assays showing HUVEC migration under TNF‐α stimulation after different treatments. Scale bar, 100 µm. (d) Quantitative analysis of migrated HUVEC numbers (*n* = 3). (e) Representative EdU staining images. Nuclei were stained with DAPI. Scale bar, 100 µm. (f) Quantitative analysis of the proportion of EdU‐positive HUVECs (*n* = 3). (g) Representative wound‐healing images showing HUVEC migration at 0 and 24 h. Scale bar, 100 µm. (h) Quantitative analysis of the relative migration area (*n* = 3). (i) ELISA quantification of VEGFA, FGF2, MMP2, and MMP9 levels in HUVECs culture supernatants after PBS or Sta@MSR treatment (*n* = 5). Data are presented as mean ± SEM. Data in (d,f,h) analyzed by one‐way ANOVA followed by Tukey's post hoc multiple‐comparison test. Data in (i) analyzed by two‐tailed Student's t‐test. ^*^ *p* < 0.05, ^**^ *p* < 0.01, ^***^ *p* < 0.001, ^****^ *p* < 0.0001.

We next investigated whether Sta@MSR could suppress inflammation‐driven endothelial activation using human umbilical vein endothelial cells (HUVECs), a well‐established in vitro model for studying angiogenesis‐related cellular behaviors. TNF‐α was employed to establish an inflammatory endothelial cell model, as this cytokine is an important mediator of endothelial activation and inflammation‐associated angiogenic responses [43]. In the Transwell migration assay, abundant migrated cells were observed in the PBS group (Figure 4c,d). Free Stattic and SR partially reduced cell migration, while Sta@SR produced a stronger inhibitory effect, indicating that STAT3 inhibition and Ru‐mediated antioxidative regulation cooperatively attenuated endothelial motility. Notably, Sta@MSR showed the most pronounced suppression of HUVEC migration. The anti‐proliferative effect of Sta@MSR was then assessed by EdU staining. Free Stattic, SR, and Sta@SR reduced endothelial proliferation to varying degrees (Figure 4e,f). Among all treatments, Sta@MSR resulted in the lowest EdU‐positive ratio, demonstrating its superior ability to inhibit endothelial proliferation under inflammatory conditions. The wound‐healing assay further confirmed the inhibitory effect of Sta@MSR on endothelial motility. PBS‐treated HUVECs rapidly migrated into the scratched area after 24 h, whereas Sta@MSR markedly delayed wound closure and maintained a larger residual scratch area than the other formulations (Figure 4g,h). To further evaluate the antiangiogenic activity of Sta@MSR, we performed a HUVEC tube formation assay. As shown in Figure S7a, HUVECs in the PBS group formed extensive, interconnected tubular networks, whereas free Stattic and SR partially inhibited network formation. Sta@SR further reduced the formation of tubular structures, with Sta@MSR exhibiting the most pronounced inhibitory effect, characterized by markedly reduced network formation and connectivity. Quantitative analysis further confirmed that Sta@MSR significantly decreased the total tube length, number of meshes, and number of junctions compared with Sta@SR (Figure S7b–d). Finally, ELISA analysis was performed to determine whether Sta@MSR could regulate the secretion of pro‐angiogenic mediators. TNF‐α‐stimulated HUVECs secreted high levels of VEGFA, fibroblast growth fator 2 (FGF2), matrix metalloproteinase 2 (MMP2), and matrix metalloproteinase 9 (MMP9) (Figure 4i). VEGFA and FGF2 are major angiogenic growth factors that promote endothelial activation and sprouting, while MMP2 and MMP9 facilitate extracellular matrix remodeling and endothelial invasion during neovessel formation [44, 45]. Sta@MSR treatment markedly reduced the secretion of these mediators.

Collectively, these results demonstrate that Sta@MSR efficiently crosses an epithelial barrier model and suppresses inflammation‐induced endothelial angiogenic responses, including migration, proliferation, and pro‐angiogenic mediator secretion. This in vitro anti‐angiogenic activity complements its antioxidative and anti‐inflammatory effects in corneal epithelial cells, supporting Sta@MSR as a multi‐level therapeutic platform for CoNV.

### 2.5 Sta@MSR Enhances Ocular Retention, Corneal Penetration and Alleviates Alkali Burn‐Induced CoNV In Vivo

Macrophage membrane cloaking endows nanocarriers with inflammation‐tropic properties by preserving membrane receptors and adhesion molecules derived from parent macrophages [46, 47]. In inflamed tissues, endothelial activation and stromal injury increase adhesion molecules such as ICAM‐1, which interact with macrophage membrane‐associated integrins, including CD11b, to promote inflammatory adhesion and lesion anchoring [31, 48]. Based on this biomimetic targeting rationale and the in vitro epithelial barrier‐crossing activity of Sta@MSR, we next evaluated its ocular retention and corneal penetration in a mouse model of alkali burn‐induced CoNV (Figure 5a). IVIS Spectrum fluorescence imaging showed that free AF488 was rapidly cleared from the ocular surface, whereas AF488@SR displayed moderately prolonged retention. Importantly, AF488@MSR maintained a stronger fluorescence signal over time than the other formulations (Figure 5b,c). Fluorescence imaging of corneal cryosections further confirmed the corneal distribution of AF488‐labeled formulations after topical administration. Compared with free AF488, AF488@SR showed increased fluorescence within corneal tissue, while AF488@MSR exhibited the strongest green fluorescence signal with clear stromal distribution (Figure 5d,e). These results suggest that Sta@MSR can effectively persist on the injured ocular surface and penetrate into the corneal stroma, enabling access to the inflammatory‐angiogenic microenvironment that drives CoNV progression.

**FIGURE 5:**
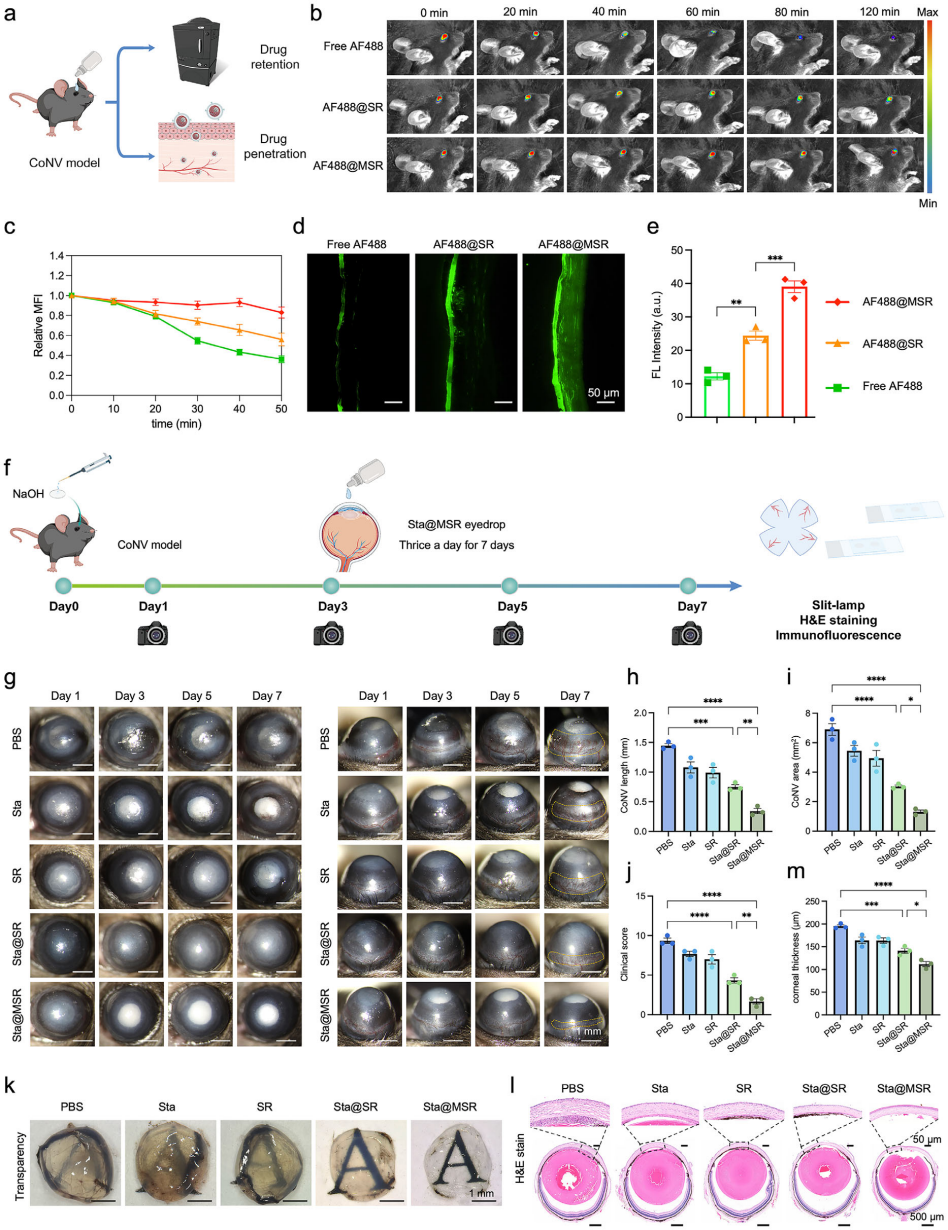
Ocular delivery performance and therapeutic efficacy of Sta@MSR in alkali burn‐induced CoNV. (a) Schematic illustration of the evaluation of ocular retention and corneal penetration of Sta@MSR after topical administration. (b) Representative in vivo fluorescence images. (c) Quantitative analysis of fluorescence intensity on the ocular surface over time (*n* = 3). (d) Representative fluorescence images of mouse corneas. Scale bar, 50 µm. (e) Quantitative analysis of corneal fluorescence intensity (*n* = 3). (f) Schematic illustration of the experimental timeline. (g) Representative slit‐lamp images in alkali‐burned mice after different treatments. Scale bar, 1 mm. (h) Quantitative analysis of CoNV length (*n* = 3). (i) Quantitative analysis of CoNV area (*n* = 3). (j) Clinical scores of mouse corneas. (*n* = 3). (k) Representative ex vivo images showing corneal transparency after different treatments. Scale bar, 1 mm. (l) H&E staining images of mouse corneas. Scale bars, 500 and 50 µm for enlarged images. (m) Quantitative analysis of corneal thickness based on H&E staining images (*n* = 3). Data are presented as mean ± SEM. Data in (e,h–j,m) analyzed by one‐way ANOVA followed by Tukey's post hoc multiple‐comparison test. ^*^ *p* < 0.05, ^**^ *p* < 0.01, ^***^ *p* < 0.001, and ^****^ *p* < 0.0001.

We then evaluated the therapeutic efficacy of Sta@MSR by administering PBS, Sta, SR, Sta@SR, or Sta@MSR topically three times daily for 7 days after alkali injury, when substantial neovascularization and persistent inflammation are evident (Figure 5f). Slit‐lamp images showed progressive corneal opacity and extensive neovascular growth in the PBS group over time (Figure 5g). Among all groups, Sta@MSR most effectively suppressed corneal neovessel ingrowth and preserved corneal clarity. Quantitative analysis confirmed that Sta@MSR significantly decreased both CoNV length and CoNV area compared with the other treatments (Figure 5h,i). Sta@MSR‐treated eyes showed the lowest clinical scores, further supporting its therapeutic benefit (Figure 5j). Consistently, ex vivo corneal transparency assessment showed that corneas from the Sta@MSR group retained the clearest appearance, allowing the underlying letter pattern to be more clearly visualized compared with the other groups (Figure 5k). Hematoxylin and eosin (H&E) staining further revealed pronounced corneal thickening, stromal edema, and structural disruption in the PBS group (Figure 5l). Although Sta, SR, and Sta@SR partially alleviated these pathological changes, Sta@MSR achieved the most effective histological protection, preserving corneal architecture and reducing inflammatory thickening. Quantitative analysis showed that Sta@MSR significantly decreased corneal thickness compared with the other groups (Figure 5m), suggesting reduced edema and tissue inflammation.

These results demonstrate that Sta@MSR exhibits favorable ocular retention and corneal penetration after topical administration, and effectively alleviates alkali burn‐induced CoNV. Its superior therapeutic efficacy is reflected by reduced neovessel length and area, improved clinical appearance, enhanced corneal transparency, and attenuated histopathological damage, supporting Sta@MSR as a promising topical nanotherapeutic platform for inflammatory CoNV.

### 2.6 Sta@MSR Remodels the Oxidative‐Inflammatory Microenvironment in Alkali‐Burned Corneas

Alkali injury initiates a self‐amplifying cascade in which excessive ROS production promotes inflammatory cytokine release, chemokine‐mediated immune‐cell recruitment, macrophage activation, and subsequent angiogenic remodeling [17, 49]. Therefore, we examined whether Sta@MSR could interrupt this oxidative‐inflammatory‐angiogenic loop in vivo. CellROX fluorescence staining revealed markedly elevated oxidative stress‐associated fluorescence in PBS‐treated corneas (Figure 6a,b). Free Stattic produced only a modest reduction in fluorescence intensity, whereas SR‐containing formulations substantially attenuated the signal. Notably, Sta@MSR exhibited the lowest CellROX fluorescence intensity among the treatment groups, suggesting the greatest reduction in corneal tissue oxidative stress. These findings support the antioxidative activity of Sta@MSR in vivo and suggest that improved ocular retention and corneal penetration may contribute to its therapeutic efficacy.

**FIGURE 6:**
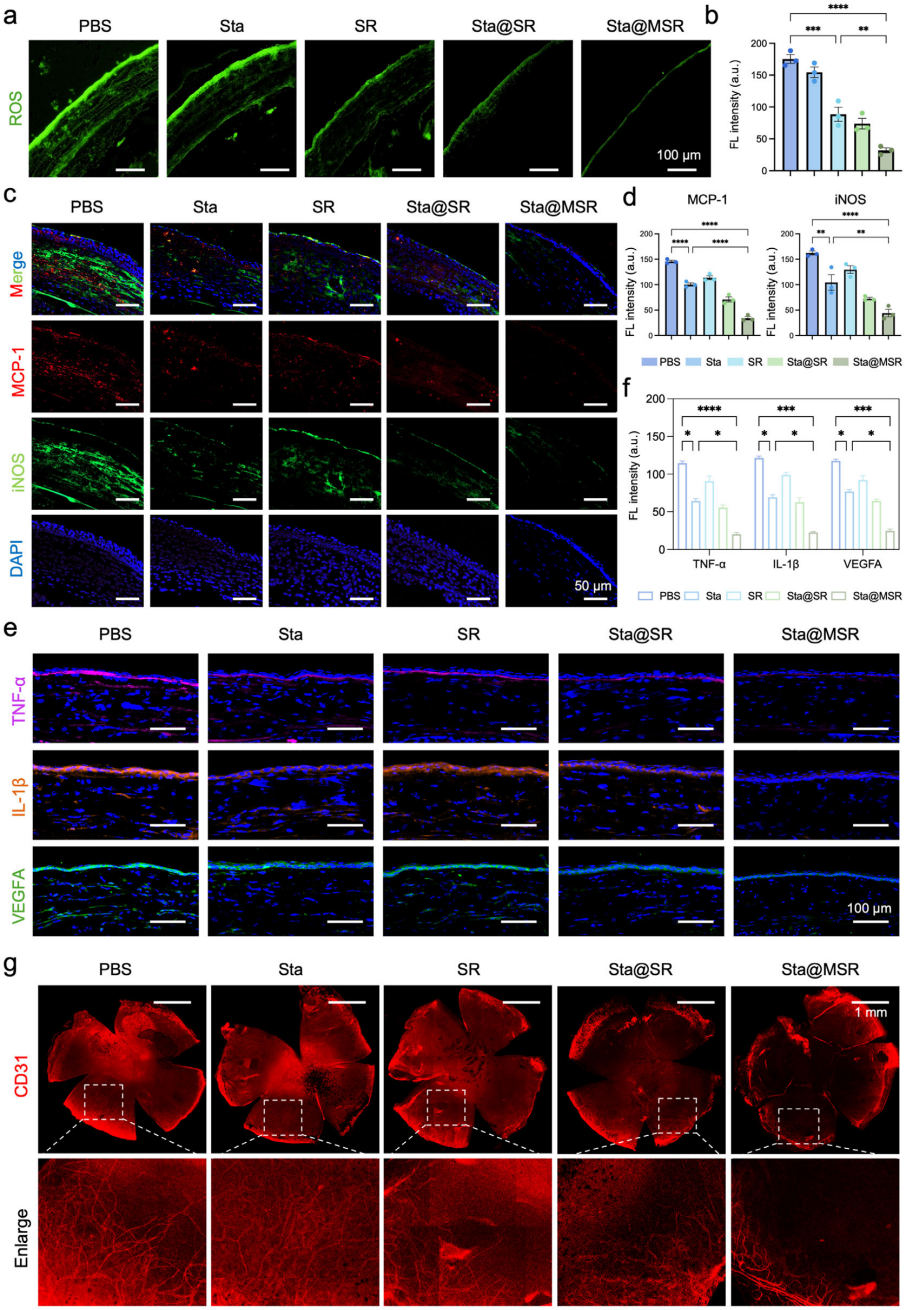
Sta@MSR suppresses oxidative stress, macrophage‐associated inflammation, and neovascularization in alkali‐burned corneas. (a) Representative CellROX fluorescence images showing oxidative stress‐associated fluorescence in alkali‐burned mouse corneas after treatment with PBS, Sta, SR, Sta@SR, or Sta@MSR. Scale bars, 100 µm. (b) Quantitative analysis of CellROX fluorescence intensity (n = 3). (c) Representative immunofluorescence images showing MCP‐1 and iNOS expression in alkali‐burned corneas after different treatments. Nuclei were stained with DAPI. Scale bar 50 µm. (d) Quantitative fluorescence intensity analysis of MCP‐1 and iNOS expression (*n* = 3). (e) Representative immunofluorescence images showing TNF‐α, IL‐1β, and VEGFA expression. Nuclei were stained with DAPI. Scale bar, 100 µm. (f) Quantitative fluorescence intensity analysis of TNF‐α, IL‐1β, and VEGFA expression (*n* = 3). (g) Representative corneal flat‐mount immunofluorescence images showing CD31‐positive neovascularization after different treatments. Scale bar, 1 mm. Data are presented as mean ± SEM. Data in (b,d,f) analyzed by one‐way ANOVA followed by Tukey's post hoc multiple‐comparison test. ^*^ *p* < 0.05, ^**^ *p* < 0.01, ^***^ *p* < 0.001, ^****^ *p* < 0.0001.

We next evaluated macrophage‐associated inflammatory responses by immunofluorescence staining of monocyte chemoattractant protein‐1 (MCP‐1) and iNOS. MCP‐1 is a key chemokine involved in monocyte/macrophage recruitment, while iNOS reflects pro‐inflammatory macrophage activation. In PBS‐treated corneas, strong MCP‐1 and iNOS signals were observed, suggesting that alkali injury induced robust chemokine production and promoted the accumulation of pro‐inflammatory macrophages within the injured corneal microenvironment (Figure 6c,d). Sta@MSR markedly suppressed both MCP‐1 and iNOS expression, indicating that it effectively blocked the chemotactic recruitment of macrophages and limited further inflammatory cell accumulation. This effect can contribute to breaking the self‐reinforcing inflammatory loop that drives persistent corneal injury and neovascularization after alkali burn. The broader inflammatory and angiogenic status of the cornea was next assessed by immunofluorescence staining of TNF‐α, IL‐1β, and VEGFA. These mediators represent interconnected components of the CoNV pathological network: TNF‐α and IL‐1β amplify inflammatory tissue injury, while VEGFA directly drives endothelial activation and neovessel formation. Free Stattic and SR reduced cytokine expression to different extents, while Sta@SR showed a more coordinated inhibitory effect. Importantly, Sta@MSR most effectively suppressed TNF‐α, IL‐1β, and VEGFA expression (Figure 6e,f).

Finally, corneal flat‐mount CD31 staining was performed to determine whether this microenvironmental remodeling translated into inhibition of pathological angiogenesis. PBS‐treated corneas displayed dense CD31‐positive vascular networks extending from the limbus toward the central cornea, indicating severe neovascular invasion (Figure 6g). Sta@MSR markedly diminished CD31‐positive neovascularization, consistent with the reduced CoNV length and area observed by slit‐lamp imaging. These findings indicate that suppression of CoNV by Sta@MSR is not an isolated anti‐angiogenic effect, but rather the downstream consequence of coordinated regulation of oxidative stress, inflammatory chemotaxis, macrophage activation, and angiogenic factor expression.

### 2.7 Sta@MSR Reshapes the Inflammatory‐Angiogenic Transcriptome and Inhibits JAK‐STAT Signaling in Alkali‐Burned Corneas

To gain a broader molecular view of how Sta@MSR remodels the injured corneal microenvironment, RNA‐seq was performed on corneas from normal mice, PBS‐treated CoNV mice, and Sta@MSR‐treated CoNV mice. The sample cluster analysis showed tight clustering of biological replicates within each group, supporting the reliability of the sequencing dataset (Figure 7a). Principal component analysis (PCA) revealed a clear separation between normal and PBS‐treated corneas, reflecting the profound transcriptional perturbation caused by alkali injury (Figure 7b). Sta@MSR‐treated samples formed a distinct cluster away from the PBS group, demonstrating that Sta@MSR substantially altered the CoNV‐associated transcriptional program. Venn analysis revealed substantial overlap among differentially expressed genes (DEGs) from the three comparisons (Figure 7c). The Normal vs. PBS comparison contained 2015 unique DEGs, indicating broad transcriptional disruption after alkali burn. Notably, 2042 DEGs were shared between the Normal vs. PBS and PBS vs. Sta@MSR comparisons, suggesting that Sta@MSR regulated many alkali burn‐responsive genes. Volcano plot analysis further confirmed extensive differential gene expression after alkali injury and marked transcriptional remodeling after Sta@MSR treatment (Figure 7d). Together, these results suggest that Sta@MSR shifts the diseased corneal transcriptome away from the untreated inflammatory state and toward a more normalized profile.

**FIGURE 7:**
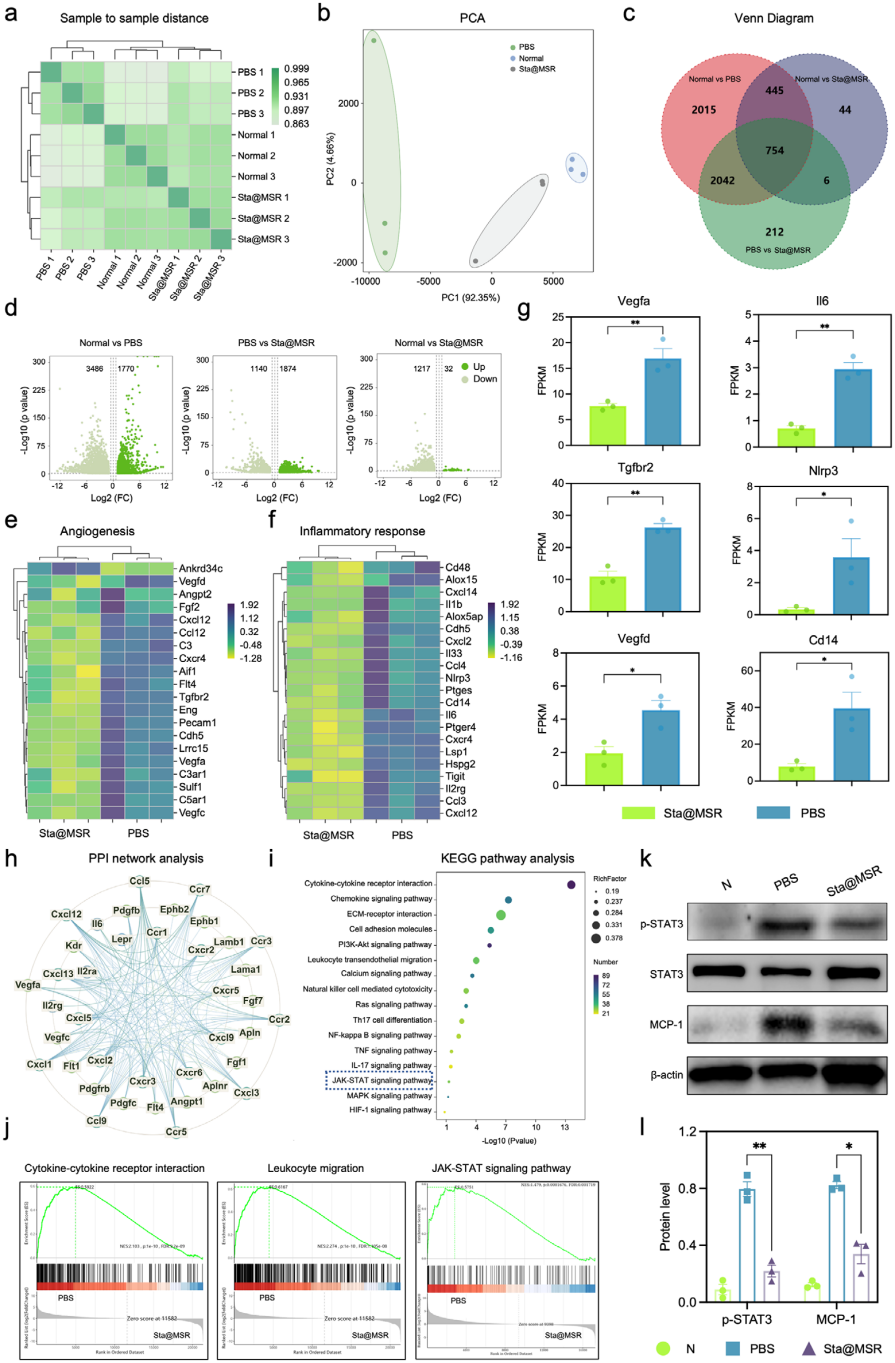
RNA‐seq analysis reveals suppression of inflammatory‐angiogenic signaling and JAK‐STAT activation by Sta@MSR. (a) Sample clustering analysis of RNA‐seq datasets from normal, PBS‐treated CoNV, and Sta@MSR‐treated CoNV corneas. (b) PCA of RNA‐seq profiles from the normal, PBS, and Sta@MSR groups. (c) Venn diagram showing overlapping and distinct DEG sets among the three comparisons. (d) Volcano plots showing differentially expressed genes among groups. (e,f) Heatmap analysis of angiogenesis‐ and inflammation‐related DEGs. (g) FPKM quantification of representative DEGs, including *Vegfa*, *Il6*, *Tgfbr2*, *Nlrp3*, *Vegfd*, and *Cd14* (*n* = 3). (h) PPI network analysis of DEGs between PBS‐ and Sta@MSR‐treated groups. (i) KEGG pathway enrichment analysis of DEGs between PBS‐ and Sta@MSR‐treated groups. (j) GSEA of cytokine‐cytokine receptor interaction, leukocyte migration, and JAK‐STAT signaling pathways. (k) Western blot analysis of p‐STAT3, STAT3, and MCP‐1 expression in normal, PBS‐treated, and Sta@MSR‐treated corneas. β‐actin was used as the loading control. (l) Quantitative analysis of p‐STAT3 and MCP‐1 protein expression. Data are presented as mean ± SEM. Statistical significance was determined by two‐tailed Student's t‐test for data g and one‐way ANOVA followed by Tukey's post hoc multiple‐comparison test for data l. ^*^ *p* < 0.05 and ^**^ *p* < 0.01.

The heatmap displays the overall pattern of DEGs among samples (Figure S8). Because angiogenesis and inflammation are central to alkali burn‐induced CoNV, we focused on these functional gene modules. Heatmap analysis showed that angiogenesis‐related genes were highly expressed in PBS‐treated corneas but markedly reduced after Sta@MSR treatment (Figure 7e). Similarly, inflammatory genes related to cytokine signaling, chemokine recruitment, inflammasome activation, and innate immunity were broadly suppressed by Sta@MSR (Figure 7f). Representative gene analysis further confirmed the downregulation of *Vegfa*, *Il6*, *Tgfbr2*, *Nlrp3*, *Vegfd*, and *Cd14* in Sta@MSR‐treated corneas (Figure 7g), indicating coordinated inhibition of angiogenic and inflammatory transcriptional programs. Protein‐protein interaction (PPI) analysis showed dense interactions among genes involved in cytokine communication, chemokine signaling, immune‐cell trafficking, inflammatory response, and angiogenesis (Figure 7h). Gene ontology (GO) enrichment analysis highlighted biological processes related to inflammatory signaling, immune regulation, cell migration, extracellular matrix organization, and angiogenesis‐associated responses (Figure S9). KEGG enrichment analysis further identified that Sta@MSR resulted in enrichment in angiogenic and inflammatory‐associated processes (such as cytokine‐cytokine receptor interaction, leukocyte transendothelial migration, and JAK‐STAT signaling) (Figure 7i). GSEA strengthened this pathway‐level interpretation. In PBS‐treated corneas, cytokine‐cytokine receptor interaction, leukocyte migration, and JAK‐STAT signaling were positively enriched, consistent with an active inflammatory‐angiogenic phenotype. Sta@MSR treatment reduced the enrichment of these pathways (Figure 7j). Western blot analysis further confirmed that alkali burn increased p‐STAT3, and MCP‐1 expression in PBS‐treated corneas, whereas Sta@MSR markedly reduced these protein levels (Figure 7k,l). These findings demonstrate that Sta@MSR inhibits JAK‐STAT3 activation, thereby reducing MCP‐1‐associated inflammatory cell recruitment in alkali‐burned corneas.

In summary, Sta@MSR reshapes the inflammatory‐angiogenic transcriptome of alkali‐burned corneas. By suppressing cytokine signaling, leukocyte migration, chemokine pathways, and JAK‐STAT activation, Sta@MSR disrupts the molecular network connecting inflammation, macrophage recruitment, and pathological neovascularization. These findings provide mechanistic support for the multi‐component design of Sta@MSR and explain its superior in vivo therapeutic efficacy in CoNV.

### 2.8 Ocular and Systemic Biosafety Evaluation of Sta@MSR

To evaluate the biosafety profile of Sta@MSR, we first examined its cytocompatibility in major corneal resident cells. Cell counting kit‐8 (CCK‐8) assays showed that Sta@MSR maintained over 80% viability in both HCECs and human corneal stromal cells (HCSCs) across the tested concentration range (Figure S10), indicating favorable corneal cytocompatibility. Furthermore, an HCEC scratch assay revealed no significant differences in wound closure between the PBS and Sta@MSR groups at either 12 or 24 h (Figure S11), suggesting that Sta@MSR does not impair epithelial wound closure under the tested conditions. Given its potential application as a topical ocular therapeutic, the in vivo biosafety of Sta@MSR was further evaluated in normal mice (Figure 8a). Body weight steadily increased in all groups, with no significant differences among mice treated with PBS, SR, Sta@SR, or Sta@MSR (Figure 8b). Intraocular pressure (IOP) remained within a normal range among all groups after treatment (Figure 8c).

**FIGURE 8:**
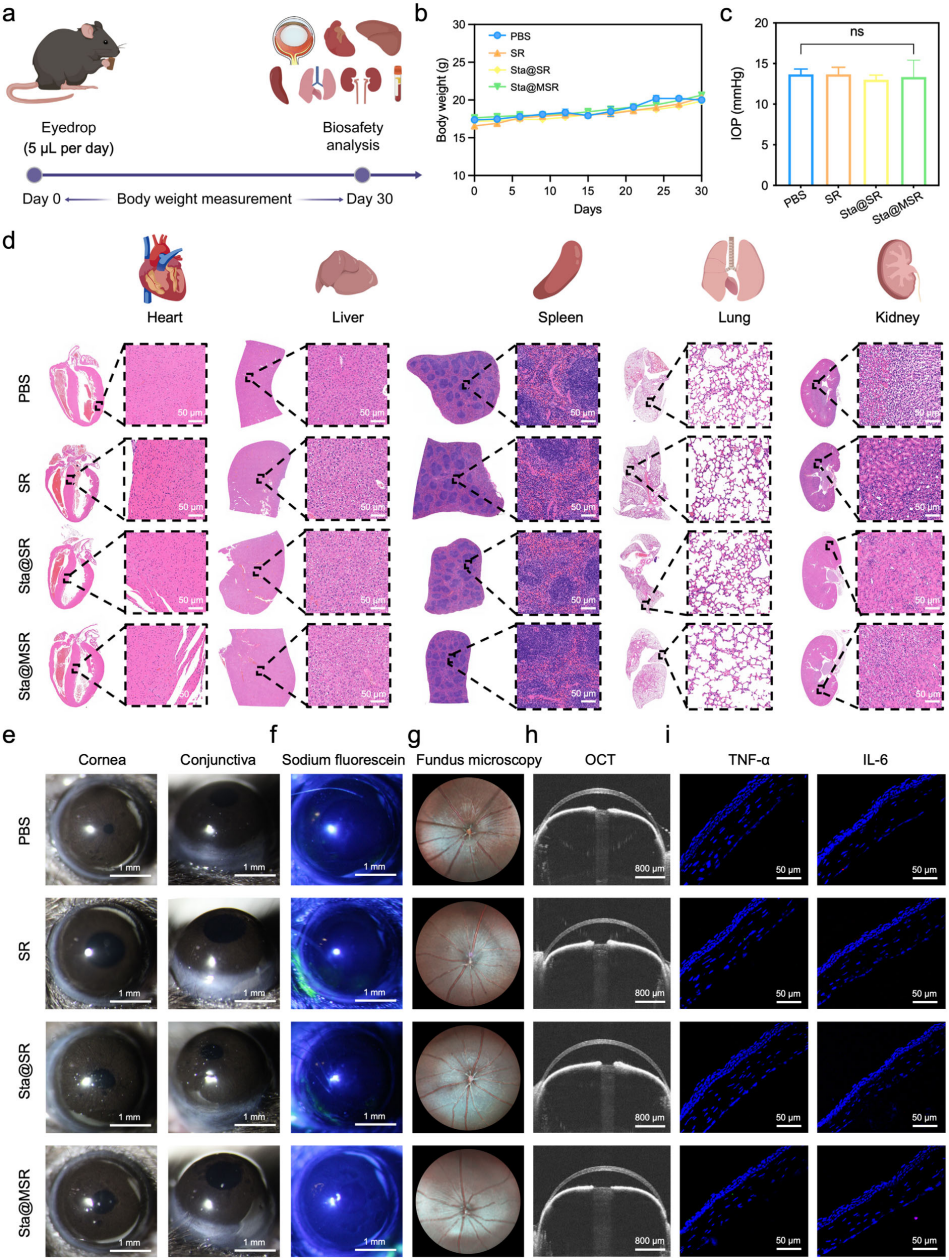
Biocompatibility of Sta@MSR in normal mice. (a) Schematic illustration of the biosafety evaluation. (b) Body weight changes of mice during the 30‐day treatment period (*n* = 3). (c) Intraocular pressure measurements after different treatments (*n* = 3). (d) H&E staining images of major organs, including the heart, liver, spleen, lung, and kidney, after different treatments. Scale bar, 50 µm. (e) Slit‐lamp images of the cornea and conjunctiva after different treatments. Scale bar, 1 mm. (f) Sodium fluorescein staining images showing corneal epithelial integrity. Scale bar, 1 mm. (g) Fundus photographs after different treatments. (h) Anterior segment OCT images. Scale bar, 800 µm. (i) Representative immunofluorescence images showing TNF‐α and IL‐6 expression in normal mouse corneas after different treatments. Nuclei were stained with DAPI. Scale bar, 50 µm. Data are presented as mean ± SEM. Data in (c) analyzed by one‐way ANOVA followed by Tukey's post hoc multiple‐comparison test.ns, not significant.

Histological analysis of major organs, including the heart, liver, spleen, lung, and kidney, showed no apparent tissue damage, inflammatory infiltration, necrosis, or structural abnormalities in Sta@MSR‐treated mice compared with the control groups (Figure 8d). These results support the systemic biocompatibility of Sta@MSR under the tested dosing regimen. Ocular safety was then examined in more detail. Slit‐lamp images showed clear corneas and intact conjunctival morphology in all groups, with no obvious signs of ocular irritation, corneal opacity, edema, or conjunctival congestion after eye‐drop administration (Figure 8e). Sodium fluorescein staining showed no apparent corneal epithelial defects in Sta@MSR‐treated eyes (Figure 8f), indicating that the formulation did not compromise epithelial integrity. Fundus photography further revealed normal retinal appearance, and optical coherence tomography (OCT) imaging showed preserved anterior segment structure without obvious morphological abnormalities (Figure 8g,h). To further assess whether Sta@MSR induced local inflammatory responses in normal corneas, immunofluorescence staining of TNF‐α and IL‐6 was performed. Only minimal fluorescence signals were observed in Sta@MSR‐treated corneas, comparable to those in the control groups (Figure 8i), suggesting that repeated topical administration did not trigger detectable corneal inflammation.

Collectively, these results establish the favorable cytocompatibility, ocular tolerance, and systemic biocompatibility of Sta@MSR after repeated topical administration. This integrated safety profile, together with its multi‐level therapeutic efficacy, supports Sta@MSR as a promising topical biomimetic nanotherapeutic platform for CoNV treatment.

## 3 Discussion

CoNV develops within an inflammatory microenvironment characterized by epithelial damage, oxidative stress, and immune‐cell infiltration, which collectively favor pathological angiogenesis [50]. Our findings identify STAT3 activation as a prominent feature of the inflammatory and angiogenic alterations associated with CoNV. In human CoNV corneas, increased STAT3 phosphorylation was accompanied by elevated VEGFA and inflammatory mediators, whereas total STAT3 expression remained unchanged. Similarly, alkali‐injured mouse corneas exhibited ROS accumulation, macrophage infiltration, enhanced STAT3 phosphorylation accompanied by pathological neovascularization. Transcriptomic analysis further revealed enrichment of JAK‐STAT signaling, chemokine signaling, and leukocyte transendothelial migration pathways. Together, these findings suggest a close association between oxidative stress, STAT3 activation, and inflammatory‐angiogenic remodeling in CoNV.

The interplay between oxidative stress and STAT3 signaling may contribute to the persistence of the inflammatory microenvironment in CoNV. Excessive ROS can modulate STAT3 activity through redox‐sensitive mechanisms, including oxidative modifications of STAT3 and protein tyrosine phosphatases [51, 52, 53, 54]. Within the ROS‐rich stroma of the alkali‐burned cornea, macrophage STAT3 would thus be held in an activated state, and its output would in turn sustain the chemokine production that recruits further macrophages and perpetuates oxidative injury [55, 56]. These interactions suggest a possible reciprocal relationship between ROS accumulation and STAT3‐associated inflammation, which contribute to sustained inflammatory and angiogenic responses following corneal alkali injury [57].

Previous studies have demonstrated the therapeutic potential of targeting oxidative stress or STAT3‐associated signaling in CoNV. Su et al. developed a Pt/Mn‐based antioxidant nanozyme that alleviated CoNV by reducing ROS accumulation and suppressing pathological angiogenesis [58]. Similarly, Zheng et al. reported that cerium oxide nanoparticles attenuated inflammation‐associated CoNV through their antioxidative activity [59]. Wu et al. further demonstrated that bufalin alleviated CoNV by modulating STAT3‐associated signaling [60]. Although these studies have established the therapeutic benefits of antioxidant treatment and STAT3‐targeted intervention, each approach primarily addresses a specific pathological component of CoNV. Antioxidant therapy can alleviate ROS‐mediated tissue injury but may not fully suppress persistent STAT3‐associated inflammatory and angiogenic signaling, while STAT3 inhibition does not directly eliminate excessive ROS accumulation. Consequently, targeting either process alone may provide incomplete regulation of the oxidative‐inflammatory microenvironment that sustains CoNV progression. To address these limitations, we developed Sta@MSR, which integrates sustained Ru‐mediated catalytic ROS scavenging with Stattic‐mediated inhibition of STAT3‐associated inflammatory and angiogenic signaling. Macrophage membrane cloaking further enhances ocular retention and corneal penetration, facilitating local therapeutic delivery. By combining these complementary functions, Sta@MSR provides an integrated strategy for suppressing oxidative stress and inflammation‐associated pathological angiogenesis in CoNV (Figure 9).

**FIGURE 9:**
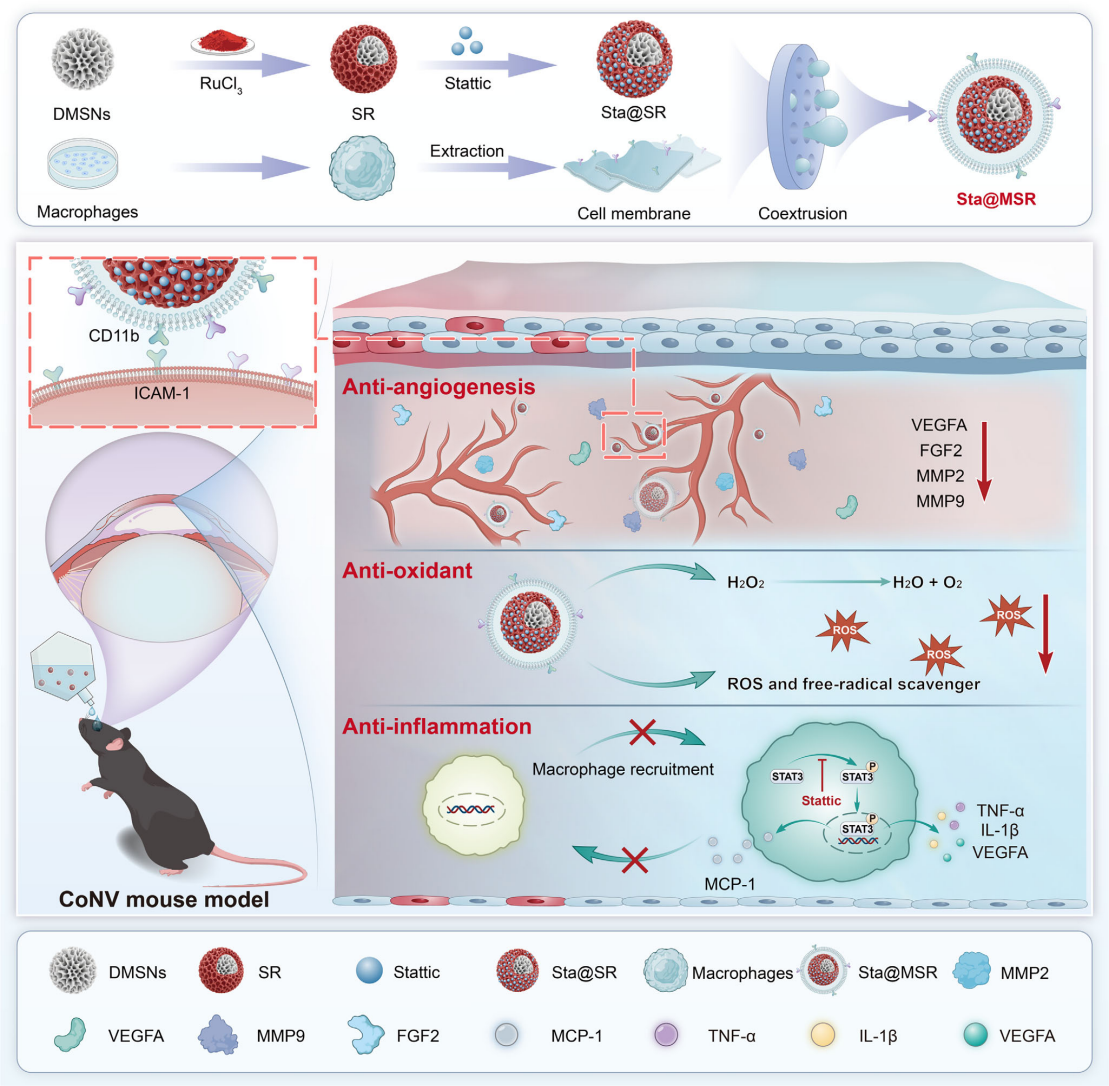
Schematic illustration of Sta@MSR construction and its therapeutic mechanism in alkali burn‐induced CoNV. Sta@MSR integrates Ru‐mediated ROS scavenging, Stattic‐mediated STAT3 inhibition, and macrophage membrane‐mediated inflammatory targeting to remodel the oxidative‐inflammatory‐angiogenic microenvironment and suppress pathological CoNV.

The therapeutic responses of the individual formulations support the advantage of this integrated strategy. Free Stattic reduced inflammatory mediator expression but showed limited effects on ROS accumulation, consistent with its role as a signaling inhibitor rather than a direct antioxidant. The SR effectively scavenged ROS but incompletely inhibited endothelial migration, proliferation, and angiogenic‐mediator secretion. By combining these functions, Sta@MSR achieved favorable suppression of oxidative stress, inflammatory signaling, and pathological neovascularization, accompanied by improved corneal transparency and reduced stromal injury. Furthermore, macrophage membrane coating enhanced ocular‐surface fluorescence retention and corneal stromal fluorescence following topical administration. This enhanced lesion accumulation is likely mediated by CD11b retained on the macrophage membrane, which engages ICAM‐1 upregulated on activated endothelial cells within CoNV lesions [31, 48]. Collectively, these results suggest that coordinated ROS scavenging and STAT3 inhibition provide more effective regulation of the inflammatory‐angiogenic microenvironment than either intervention alone.

Several limitations of this study should be acknowledged. Although our findings support an association among oxidative stress, STAT3 activation, macrophage‐associated inflammation, and angiogenesis, the causal relationships and cell‐specific contributions of these processes remain to be fully established. In addition, longer‐term investigations of ocular pharmacokinetics, Ru distribution and clearance, and safety in larger animal models are warranted. Despite these limitations, our findings support a microenvironment‐remodeling strategy for CoNV that combines redox regulation with suppression of inflammatory STAT3‐associated signaling. Sta@MSR therefore provides a topical proof‐of‐concept platform for further development against ocular surface diseases involving coupled oxidative stress, inflammation, and pathological angiogenesis.

## 4 Experimental Section

### 4.1 Synthesis of DMSNs

DMSNs were synthesized by a cetyltrimethylammonium bromide (CTAB)‐assisted sol‐gel co‐condensation method. Briefly, CTAB was dissolved in a mixed solvent containing deionized water, ethanol, and ammonia solution under stirring, followed by the addition of diethyl ether to form a homogeneous reaction system. After the surfactant template was fully assembled, tetraethyl orthosilicate (TEOS) and (3‐aminopropyl)triethoxysilane (APTES) were added dropwise under continuous stirring. TEOS served as the silica precursor, while APTES was co‐condensed with TEOS to introduce amino groups into the silica framework. The reaction was maintained at 60°C for 72 h to form amino‐functionalized DMSNs. The resulting nanoparticles were collected by centrifugation at 12 000 rpm for 10 min and washed three times with anhydrous ethanol. To remove the CTAB template, the nanoparticles were refluxed in an acidic ethanol solution (1% HCl in ethanol, v/v) at 60°C for 6 h, followed by thorough washing with ethanol and deionized water. Finally, the obtained DMSNs were dried under vacuum at 60°C overnight.

### 4.2 Synthesis of SR

SR was prepared by in situ adsorption and reduction of Ru species on amino‐functionalized DMSNs. Briefly, DMSNs (10 mg) were dispersed in an aqueous RuCl_3_·xH_2_O solution (5 mg/mL, 5 mL) and stirred at room temperature for 12 h to allow Ru ions to adsorb onto the amino‐functionalized silica framework. Ru^3^^+^ was then reduced by adding freshly prepared NaBH_4_ solution containing NaBH_4_ (1 mg), followed by continuous stirring for another 2 h. The resulting SR nanoparticles were collected by centrifugation at 12 000 rpm for 10 min and washed three times with deionized water to remove unbound Ru species. The obtained SR nanoparticles were stored in deionized water at 4°C until further use.

### 4.3 Preparation of Sta@SR

Sta@SR was prepared by passive diffusion of Stattic into the porous SR framework. Briefly, SR nanoparticles (5 mg) were dispersed in Stattic solution (1 mg/mL) and stirred at room temperature for 24 h under light‐protected conditions. Unloaded Stattic was removed by centrifugation at 12 000 rpm for 10 min, followed by three washes with PBS. The Stattic EE and LC of Sta@SR were determined via HPLC analysis and calculated using the following formula:

(1) $$\begin{array}{c} \mathrm{EE}\;(\%)=\frac{W_{\mathrm{loaded}\;\mathrm{Stattic}}}{W_{\mathrm{initial}\;\mathrm{Stattic}}}\times 100\%\; \end{array}$$

(2) $$\begin{array}{c} \mathrm{LC}\;(\%)=\frac{W_{\mathrm{loaded}\;\mathrm{Stattic}}}{W_{\mathrm{Sta}@\mathrm{SR}}}\times 100\%\; \end{array}$$

### 4.4 Macrophage Membrane Extraction and Preparation of Sta@MSR

RAW264.7 macrophages were selected as the membrane source because their stable culture and expansion under standardized conditions facilitate reproducible membrane preparation and minimize variability associated with primary macrophage isolation. Macrophage membranes were extracted from RAW264.7 cells according to a previously reported method with minor modifications [33]. Briefly, RAW264.7 cells were washed with cold PBS, and the cell pellets were dispersed in homogenization buffer. The suspension was transferred into a glass homogenizer, and the cells were mechanically disrupted by 15–20 strokes. The homogenate was then centrifuged at 2000 rpm for 10 min to remove large cell debris and nuclei. The collected supernatant was further centrifuged at 20 000 rpm for 30 min, after which the supernatant was discarded, and the macrophage membrane was collected. The membrane fraction was washed with PBS and resuspended in PBS for subsequent coating.

To prepare Sta@MSR, macrophage membrane vesicles were mixed with Sta@SR nanoparticles at a 1:1 wt ratio. The mixture was sonicated in an ice bath for 2 min and subsequently extruded through a 200 nm polycarbonate membrane for 11 passes using a mini extruder. The obtained macrophage membrane‐coated Sta@SR nanoparticles were denoted as Sta@MSR.

### 4.5 Clinical Sample Acquisition

Corneal tissues were collected from patients with confirmed CoNV diagnosis who underwent corneal transplantation at Shandong Eye Hospital. Corneal stromal lenticules served as controls and were obtained from patients undergoing femtosecond laser‐assisted refractive surgery with no concurrent ocular pathologies. All tissue collection and experimental procedures were conducted with the approval of the Ethics Committee of Shandong Eye Hospital (Approval No. R202402270049).

### 4.6 Animals

C57BL/6 female mice (6–8 weeks old) were purchased from Jinan Pengyue Laboratory Animal Breeding Co., Ltd. (Jinan, China) and housed in a specific pathogen‐free (SPF) environment under a 12 h light/dark cycle with ad libitum access to food and water for one week before experiments. All animal experiments were approved by the Animal Ethics Committee of Shandong Eye Institute and complied with the Association for Research in Vision and Ophthalmology (ARVO) Statement for the Use of Animals in Ophthalmic and Vision Research (Approval No. W2024022700018).

### 4.7 Wound Healing Assay

HUVECs were seeded in 6‐well plates (1 × 10^5^ cells per well) and grown to full confluency. Cells were pre‐stimulated with TNF‐α (10 ng/mL) for 12 h to establish an inflammatory state. A linear scratch wound was then introduced using a sterile 200 µL pipette tip, and detached cells were removed by washing twice with PBS. Then, the cells were treated under different conditions and the images were acquired at 0 and 24 h. Wound migration area was quantified using ImageJ software. For HCEC scratch wound‐healing assay, HCECs were cultured to form a confluent monolayer, followed by the introduction of a linear scratch using a sterile pipette tip. After washing with PBS to remove detached cells, HCECs were treated with PBS or Sta@MSR. Images were acquired at 0, 12, and 24 h, and the wound closure area was quantified using ImageJ software.

### 4.8 Tube Formation Assay

Matrigel (Corning, USA) was thawed overnight at 4°C and kept on ice before use. A pre‐chilled 96‐well plate was coated with 50 µL Matrigel per well and incubated at 37°C for 30 min to allow gel polymerization. Following the treatments described above, HUVECs were collected and seeded into Matrigel‐coated wells at a density of 3 × 10^4^ cells per well in 100 µL of culture medium. After incubation for 6 h, tubular networks were imaged using an inverted microscope. Total tube length, number of meshes, and number of junctions were quantified using the Angiogenesis Analyzer plugin in ImageJ software (National Institutes of Health, USA).

### 4.9 Clinical Assessment

Alkali‐burned mice were randomly assigned to five groups: PBS, Stattic, SR, Sta@SR, and Sta@MSR (Stattic was normalized to 300 µg/mL). Eye drops (5 µL per eye) were administered three times daily for 7 days. Slit‐lamp images were captured at days 1, 3, 5, and 7 using the Topcon SL‐D701 Slit Lamp Microscope (Topcon, Japan). CoNV length, CoNV area, and clinical scores were assessed by two independent masked observers. Corneal injury scores and neovascularization were assessed according to established methods [61]. The calculation formula for CoNV is as follows:

(3) $$S=\frac{C}{12}\times 3.1416\times \left[R^{2}-{\left(R-L\right)}^{2}\right]$$

where S represents the area of CoNV, C represents the number of clock hours covered by CoNV, R represents the corneal radius, and L represents the maximum length of neovascularization.

### 4.10 Statistical Analysis

All data are presented as the mean ± standard error of the mean (SEM). Statistical analyses were performed using GraphPad Prism (version 9.0, GraphPad Software, La Jolla, CA, USA). Comparisons between two groups were performed using a two‐tailed Student's t‐test. Multiple group comparisons were assessed by ANOVA followed by Tukey's post hoc multiple‐comparison test. A p‐value of < 0.05 was considered statistically significant. Statistical significance was indicated as ^*^ *p* < 0.05, ^**^ *p* < 0.01, ^***^ *p* < 0.001, and ^****^ *p* < 0.0001; ns indicates no significant difference.

Other materials and methods are presented in the Supplementary Information.

## Author Contributions

Q.L., R.L. and B.R. performed the conceptualization, methodology, investigation, and original draft writing. W.L. and H.G. conceived and supervised the study, acquired funding, and revised the manuscript. C.G., H.Y., J.C., B.F., C.W., Q.B., X.L. and Y.L. performed the methodology, software, investigation, validation and formal analysis. All authors read and approved the final manuscript.

## Ethics Statement

The collection and use of human corneal tissues were approved by the Ethics Committee of Shandong Eye Hospital (Approval No. R202402270049). All animal experiments were approved by the Animal Ethics Committee of Shandong Eye Institute (Approval No. W2024022700018) and conducted in accordance with the Association for Research in Vision and Ophthalmology (ARVO) Statement for the Use of Animals in Ophthalmic and Vision Research.

## Conflicts of Interest

The authors declare no conflicts of interest.

## Supporting information




**Supporting File**: advs78647‐sup‐0001‐SuppMat.docx.

## Data Availability

The data that support the findings of this study are available from the corresponding author upon reasonable request.
