# Supplementary material for: Dual Suppression of Oxidative Stress and JAK/STAT3 Signaling Attenuates Inflammation‐Driven Corneal Neovascularization
Source: Adv Sci (Weinh). 2026 Nov 10:e78647. Online ahead of print. doi: 10.1002/advs.78647 (PMC13749474; doi:10.1002/advs.78647)
Supplement: Supplementary file 1 [file ADVS-9999-e78647-s001.docx]

Supplementary Information for

Dual Suppression of Oxidative Stress and JAK/STAT3 Signaling Attenuates Inflammation-Driven Corneal Neovascularization

Qinghua Li^1,2,3^, Ruoxi Li^1,2,3^, Bingxin Ren^1,2,3^, Chaoya Gong^3^, Hui Yan^3^, Jie Chen^3^, Benzhao Fu^3^, Chaoqun Wei^1,2,3^, Qingdong Bao^1,2,3^, Yang Li^4^, Xiaoxue Liu^1,2,3*^, Wenlong Li^1,2,3*^, and Hua Gao^1,2,3*^

Correspondence: Xiaoxue Liu (xxliu@email.sdfmu.edu.cn); Wenlong Li (wlli@sdfmu.edu.cn); Hua Gao (hgao@sdfmu.edu.cn)

**This PDF file includes:**

Materials and Methods

Figures S1 to S14

**Materials and Methods**

**Materials**

Hexadecyltrimethylammonium bromide (CTAB), tetraethyl orthosilicate (TEOS), (3-aminopropyl)triethoxysilane (APTES), 1,1-diphenyl-2-picrylhydrazyl (DPPH), and ruthenium(III) chloride hydrate (RuCl₃·xH₂O) were purchased from Sigma-Aldrich Trading Co., Ltd. (Shanghai, China). Stattic, sodium fluorescein, lipopolysaccharide (LPS), and tumor necrosis factor-α (TNF-α) were obtained from MedChemExpress, LLC. (New Jersey, USA). Phosphate-buffered saline (PBS), 4% paraformaldehyde, DAPI, goat serum, Triton X-100, and BeyoClick EdU-488 Cell Proliferation Kit were purchased from Beyotime Biotechnology Co., Ltd. (Shanghai, China). CellROX™ Reagent for Oxidative Stress Detection was purchased from Thermo Fisher Scientific (Waltham, MA, USA). Hematoxylin and eosin (H&E) staining solution was obtained from Beijing Solarbio Science & Technology Co., Ltd. (Beijing, China). Tissue-Tek® O.C.T. Compound was obtained from Sakura Finetek Co., Ltd. (Osaka, Japan). Fetal bovine serum (FBS), Dulbecco’s modified Eagle’s medium (DMEM), and DMEM/F12 were purchased from Gibco (Thermo Fisher Scientific, USA). Endothelial cell medium (ECM) was purchased from ScienCell Research Laboratories (Carlsbad, CA, USA). Matrigel was purchased from Corning Inc. (NY, USA). Alexa Fluor 488 NHS Ester (AF488), the bicinchoninic acid (BCA) protein assay kit, and PVDF membranes were purchased from Thermo Fisher Scientific (Waltham, MA, USA). SDS-PAGE gels were purchased from Epizyme Biomedical Technology Co., Ltd. (Shanghai, China). The Enhanced CCK-8 Kit was purchased from SparkJade Biotechnology Co., Ltd. (Jinan, China). Primary antibodies against TNFR1, CXCR2, TNF-α, IL-1β, IL-6, iNOS, GAPDH, β-actin, and CD11b were purchased from Proteintech Group, Inc. (Wuhan, China). CD31, VEGFA, F4/80, p-STAT3, STAT3, and MCP-1 antibodies were purchased from Abcam (Cambridge, UK). ELISA kits for VEGFA, TNF-α, IL-1β, IL-6, FGF2, MMP2, and MMP9 were purchased from Shanghai Jianglai Biotechnology Co., Ltd. (Shanghai, China). All other reagents were of analytical grade and used without further purification.

**Characterization of Sta@MSR**

Nanoparticle morphology was examined by transmission electron microscopy (TEM; HT7800, Hitachi, Japan) at an accelerating voltage of 120 kV. High-resolution images and EDS elemental mapping were obtained using a Talos F200i high-resolution TEM (Thermo Fisher Scientific, USA) at 200 kV. Hydrodynamic diameter, polydispersity index (PDI), and zeta potential were measured by dynamic light scattering (DLS) using a Malvern NanoZS90 (Malvern Instruments, UK) at 25 °C. Colloidal stability was assessed by monitoring hydrodynamic size of Sta@MSR over 7 days in deionized water, PBS, and complete cell culture medium (containing 10% FBS) at 37 °C. XPS analysis was performed using a K-Alpha X-ray photoelectron spectrometer (Thermo Fisher Scientific, USA) with Al Kα radiation. Western blot was conducted to verify membrane protein retention, with protein separation by SDS-PAGE (Epizyme, China) and chemiluminescence imaging using a Bio-Rad ChemiDoc XRS+ system (Bio-Rad, USA).

**DPPH Radical Scavenging Assay**

For the concentration-dependent assay, DPPH solution (1 mM in ethanol) was mixed with an equal volume of DMSNs, Sta@SR, or Sta@MSR suspensions at different concentrations (0-500 μg/mL). After incubation in the dark at room temperature for 30 min, the absorbance was measured at 517 nm. For the time-dependent assay, nanoparticle suspensions at a fixed concentration were mixed with DPPH solution under the same conditions, and the absorbance at 517 nm was recorded at 0, 1, 3, 5, 15, 30, 45, and 60 min. The DPPH scavenging efficiency was calculated as follows:

$$\begin{aligned} \text{Scavenging}\text{ }\text{efficiency}\text{ }\text{(\%)}\text{ }\text{=}\text{ }\left[ \text{1-}\frac{\text{A}_{\text{S}}\text{-}\text{A}_{\text{C}}}{\text{A}_{\text{B}}} \right]\text{×100\%}\text{ }\#(\text{1)} \end{aligned}$$

A_S_ is the absorbance of the mixture containing both sample and DPPH, A_C_ is the absorbance of the sample solution without DPPH, and A_B_ is the absorbance of DPPH solution without sample.

For UV-vis absorption spectroscopy analysis, Sta@MSR suspensions at different concentrations were mixed with DPPH solution and incubated in the dark at room temperature for 30 min. UV-vis absorption spectra were recorded from 500 to 600 nm. For electron paramagnetic resonance (EPR) analysis, the mixtures were transferred into capillary tubes, and the characteristic DPPH• signals were recorded using an EPR spectrometer.

**H_2_O_2_ Scavenging Assay**

For the concentration-dependent assay, H_2_O_2_ solution (100 mM in deionized water) was mixed with an equal volume of DMSNs, Sta@SR, or Sta@MSR suspensions at different concentrations (0-500 μg/mL). After incubation in the dark at room temperature for 30 min, generated bubbles were removed by gentle shaking, and the absorbance was measured at 240 nm. For the time-dependent assay, nanoparticle suspensions at a fixed concentration were mixed with H_2_O_2_ solution, and the absorbance at 240 nm was recorded at 0, 1, 3, 5, 15, 30, 45, and 60 min. The H₂O₂ scavenging efficiency was calculated using Eq. (1). A_S_ is the absorbance of the mixture containing both sample and H_2_O_2_, A_C_ is the absorbance of the sample solution without H_2_O_2_, and A_B_ is the absorbance of H_2_O_2_ solution without sample.

**Recyclable Scavenging Assay**

The recyclable scavenging activity of Sta@MSR was evaluated using repeated DPPH and H_2_O_2_ scavenging assays. Briefly, Sta@MSR suspension was mixed with DPPH solution or H_2_O_2_ solution and incubated under the same conditions described above. After each cycle, the mixture was centrifuged, the supernatant was discarded, and the recovered Sta@MSR was washed and resuspended in fresh DPPH or H_2_O_2_ solution. The same procedure was repeated until the scavenging activity could no longer be maintained. The absorbance was recorded at 517 nm for DPPH and 240 nm for H_2_O_2_, respectively.

**Cell Culture**

Human corneal epithelial cells (HCECs), human corneal stromal cells (HCSCs), human umbilical vein endothelial cells (HUVECs) were purchased from the ScienCell Research Laboratories (Carlsbad, CA, USA), and RAW264.7 macrophages were obtained from the American Type Culture Collection (ATCC) (Manassas, VA, USA). All cell lines were confirmed contamination-free by mycoplasma PCR testing and STR profiling. HCECs and HCSCs were cultured in DMEM/F12, and RAW264.7 cells were cultured in high-glucose DMEM. Both media were supplemented with 10% FBS, 100 IU/mL penicillin, and 100 μg/mL streptomycin. HUVECs were maintained in endothelial cell medium according to the manufacturer’s instructions. All cells were maintained at 37 °C in 5% CO_2_.

**Cellular Uptake Assay**

SR nanoparticles were labeled with Alexa Fluor 488 NHS Ester. Briefly, SR nanoparticles were reacted with Alexa Fluor 488 NHS Ester for 2 h at room temperature in the dark. AF488@SR was further coated with macrophage membranes using the same membrane-cloaking procedure described above to obtain AF488@MSR. Then, HCECs were seeded in 12-well plates (1.5 × 10^5^ cells per well) and cultured for 24 h at 37 °C in 5% CO_2_. To establish an inflammatory epithelial cell model, HCECs were pre-stimulated with LPS (10 μg/mL) for 12 h. Cells were then incubated with free AF488, AF488@SR, and AF488@MSR for 2 h. After three PBS washes, cells were fixed with 4% paraformaldehyde (Beyotime Biotechnology, China) for 20 min at room temperature. F-actin was stained with rhodamine phalloidin for 60 min, followed by nuclear staining with DAPI for 10 min. Fluorescence images were acquired using a confocal laser scanning microscope (LSM 980, ZEISS, Germany). For quantitative analysis, cells were resuspended in PBS and analyzed using a CytoFLEX flow cytometer (Beckman Coulter, USA). For time- and concentration-dependent uptake analysis, LPS-stimulated HCECs were incubated with AF488@MSR for different time periods or treated with different concentrations of AF488@MSR.

**Intracellular Oxidative Stress Detection**

Intracellular oxidative stress was evaluated using CellROX™ Reagent (Thermo Fisher Scientific, USA). HCECs were plated at 2 × 10^6^ cells per well in 6-well plates. After 12 h, cells were exposed to H_2_O_2_ (500 μM) in serum-free medium for 6 h. Following stimulation, cells were treated with PBS, Stattic, SR, Sta@SR, or Sta@MSR for 6 h. Cells were subsequently incubated with CellROX reagent at a final concentration of 5 μM for 15 min at 37 °C in the dark, washed with PBS, and imaged using a confocal laser scanning microscope. Fluorescence intensity was quantified using ImageJ software.

**Cellular Immunofluorescence**

HCECs were seeded in 6-well plates (2 × 10^6^ cells per well) and stimulated with LPS (10 μg/mL) for 12 h to induce a pro-inflammatory state. Cells were then treated with PBS, Stattic, SR, Sta@SR, or Sta@MSR for 12 h. Cells were fixed with 4% paraformaldehyde for 10 min at room temperature and blocked with goat serum (Beyotime Biotechnology, China) for 1 h. Cells were incubated overnight at 4 °C with primary antibodies against IL-6 (1:300), TNF-α (1:500), and IL-1β (1:300). After three PBS washes, cells were incubated with Alexa Fluor 594-conjugated secondary antibodies (1:500) for 1 h at room temperature, and nuclei were counterstained with DAPI (Beyotime Biotechnology, Shanghai, China). Images were acquired using the ZEISS LSM 900 confocal microscope and fluorescence intensity was quantified using ImageJ.

**Penetration Assay**

Epithelial barrier penetration was assessed using a transwell co-culture model. HCECs (1 × 10^5^ cells) were seeded in the upper chamber and cultured until formation of a confluent monolayer. HUVECs (1 × 10^5^ cells) were seeded on the lower chamber. Then, free AF488, AF488@SR, and AF488@MSR were added to the upper chamber and incubated for 12 h. After three PBS washes, lower-chamber inserts were fixed with 4% paraformaldehyde for 30 min, stained with DAPI for 30 min, and imaged by confocal laser scanning microscope (ZEISS LSM 900, Germany).

**Transwell** **Migration Assay**

HUVECs were pre-stimulated with TNF-α (10 ng/mL) for 12 h to establish an inflammatory state and then treated with PBS, free Stattic, SR, Sta@SR, or Sta@MSR. After treatment, HUVECs were seeded into the upper chambers of Transwell inserts with 8 μm pore-size membranes at a density of 4 × 10^4^ cells per insert. The lower chambers were filled with culture medium containing 10% FBS to induce cell migration. After 24 h, non-migrated cells on the upper surface of the membrane were gently removed with a cotton swab. Migrated cells on the lower surface were fixed with 4% paraformaldehyde and stained with crystal violet. Images were acquired using an inverted microscope, and the number of migrated cells was quantified using ImageJ software.

**EdU Proliferation Assay**

HUVECs were seeded in glass-bottom cell culture dishes (1 × 10^5^ cells per dish) and pre-stimulated with TNF-α (10 ng/mL) for 12 h to establish an inflammatory state. Then, cells were treated with various formulations. After 24 h, medium was replaced with fresh medium containing EdU (10 μM) and incubated at 37 °C for 4 h. Cells were fixed with 4% paraformaldehyde for 15 min, washed three times with PBS, and processed using a BeyoClick EdU-488 Cell Proliferation Kit (Beyotime Biotechnology, China) per the manufacturer's instructions. After DAPI counterstaining, images were acquired by CLSM (ZEISS LSM 900, ZEISS, Germany).

**Enzyme-Linked Immunosorbent Assay (ELISA)**

For in vitro analysis, HUVECs were pre-stimulated with TNF-α (10 ng/mL) for 12 h and then treated with PBS, free Stattic, SR (100 μg/mL), Sta@SR (100 μg/mL), or Sta@MSR (100 μg/mL) for 24 h. Culture supernatants were collected and centrifuged at 1,000 × g for 5 min at 4 °C to remove cell debris. VEGFA, FGF2, MMP2, and MMP9 levels in the supernatants were measured using ELISA kits according to the manufacturer’s instructions. For in vivo analysis, mouse corneas harvested on day 7 were homogenized in PBS, and the levels of VEGFA, TNF-α, IL-1β, and IL-6 were measured using ELISA kits according to the manufacturer’s instructions.

**Alkali Burn-Induced CoNV Model**

Mice underwent general anesthesia via intraperitoneal injection of Telazol (60 mg/kg) and xylazine (6 mg/kg), followed by local application of 0.5% proparacaine to anesthetize the corneal surface of the right eye. Under topical anesthesia, a circular filter paper disc (diameter 2 mm) saturated with 1 N NaOH was applied to the central cornea of the right eye for 30 s, followed by immediate irrigation with 20 mL of sterile saline. Tobramycin eye drops (0.3%) were applied post-injury to prevent infection.

**In Vivo Ocular Retention and Corneal Penetration**

Ocular surface retention was evaluated in alkali-burned mice (n = 3 per group). Free AF488, AF488@SR, and AF488@MSR (5 μL per eye) were instilled topically, and fluorescence intensity was imaged at 0, 20, 40, 60, 80, and 120 min post-instillation using an IVIS Lumina imaging system (PerkinElmer, USA). For corneal penetration, we administered free AF488, AF488@SR, and AF488@MSR onto the corneal surface. The eyeballs were collected and embedded in Tissue-Tek® OCT compound (SAKURA, Japan). Cryosections (4 μm) were prepared using a CryoStar NX50 cryostat (Thermo Fisher Scientific, USA), and imaged using the ZEISS LSM 900 confocal microscope. Corneal fluorescence intensity was quantified using ImageJ.

**Histological Assessment**

For ex vivo transparency assessment, enucleated eyes were placed over a printed letter pattern and photographed under standardized lighting. For histological analysis, corneas were embedded in OCT compound, cryosectioned at 4 μm, and stained with H&E (Solarbio, Beijing, China). Histological images were taken using a Leica DMi8 (Germany). Corneal thickness was quantified from H&E sections using ImageJ software.

**Immunofluorescence Staining**

Mouse corneal cryosections (4 μm) were fixed with 4% paraformaldehyde for 15 min and permeabilized with 0.3% Triton X-100 for 10 min. After blocking with goat serum for 1 h at room temperature, sections were incubated overnight at 4 °C with the indicated primary antibodies at appropriate dilutions, including anti-CD31 (1:200), anti-F4/80 (1:200), anti-p-STAT3 (1:200), anti-VEGFA (1:200), anti-TNF-α (1:500), anti-IL-1β (1:300), anti-iNOS (1:200), and anti-MCP-1 (1:200). After three washes with PBS, sections were incubated with corresponding Alexa Fluor-conjugated secondary antibodies for 2 h at room temperature in the dark. Nuclei were counterstained with DAPI. Images were acquired using a ZEISS LSM 900 confocal microscope, and fluorescence intensity was quantified using ImageJ software.

**Corneal Tissue Oxidative Stress Detection**

Corneal tissue oxidative stress was assessed using CellROX™ Reagent. Corneal sections were incubated with CellROX reagent for 20 min at 37 °C in the dark, washed twice with PBS, and immediately imaged using the Olympus IX73 inverted fluorescence microscope (Olympus, Japan). Fluorescence intensity was quantified using ImageJ.

**Corneal Flat Mount CD31 Immunostaining**

Harvested corneas were fixed in a 1:1 mixture of ice-cold acetone and methanol at room temperature for 15 min. After washing with PBS, corneas were blocked with 5% donkey serum containing 0.3% Triton X-100 at 37 °C for 1.5 h and incubated overnight at 4 °C with anti-CD31 antibody (10 μg/mL). After three washes with PBS, corneas were incubated with rhodamine-conjugated donkey anti-goat IgG secondary antibody (1:50) at 37 °C for 1 h in the dark. The corneas were then cut into four quadrants, flat-mounted on glass slides, and coverslipped with anti-fade mounting medium containing DAPI. Images were captured using a ZEISS LSM 900 confocal microscope.

**Western Blot**

Corneal tissues or cells were homogenized in RIPA lysis buffer supplemented with PMSF and a protease and phosphatase inhibitor cocktail. Protein concentrations were determined using a BCA protein assay kit. Equal amounts of protein (30 μg per lane) were separated by SDS-PAGE and transferred onto PVDF membranes. The membranes were blocked with quick blocking buffer for 20 min at room temperature and incubated overnight at 4 °C with the indicated primary antibodies: anti-VEGFA (1:3000), anti-iNOS (1:2000), anti-TNF-α (1:1000), anti-IL-1β (1:3000), anti-IL-6 (1:1000), anti-p-STAT3 (1:2000), anti-STAT3 (1:2000), anti-MCP-1 (1:2000), anti-β-actin (1:20000), anti-TNFR1 (1:1000), anti-CXCR2 (1:1000), anti-CD11b (1:1000), and anti-GAPDH (1:30000). After washing with TBST, the membranes were incubated with HRP-conjugated secondary antibodies for 1 h at room temperature. Protein bands were visualized using a Bio-Rad ChemiDoc XRS+ imaging system, and densitometric analysis was performed using ImageJ. The uncropped original WB image is shown in Figure S12-14.

**RNA Sequencing**

Corneas were collected from mice with alkali burn-induced CoNV that received PBS or Sta@MSR treatment. Corneas from untreated normal mice served as the control group. Total RNA was extracted from the corneas using TRIzol® Reagent according to the manufacturer’s instructions (Magen). The mRNA was purified from 1 μg of total RNA using oligo (dT) magnetic beads followed by fragmentation carried out using divalent cations at elevated temperatures in ABclonal First Strand Synthesis Reaction Buffer. Using the mRNA fragments as templates, double-stranded cDNA fragments were synthesized and connected with adapter sequences for PCR amplification. Finally, sequencing was performed with an Illumina Novaseq 6000 /MGISEQ-T7 instrument. The data generated from Illumina/BGI platform were used for bioinformatics analysis. All of the analyses were performed using an in-house pipeline from Shanghai Applied Protein Technology.

**Cell Viability Assay**

The cytocompatibility of Sta@MSR was evaluated using an Enhanced CCK-8 Kit. HCECs and HCSCs were seeded in 96-well plates at a density of 8 × 10^3^ cells per well and cultured for 24 h. Cells were then incubated with Sta@MSR at different concentrations for 24 h. After treatment, CCK-8 reagent diluted 1:9 in culture medium was added to each well, followed by incubation at 37 °C in a humidified atmosphere containing 5% CO_2_ for 4 h under light-protected conditions. The absorbance at 450 nm was measured using a Varioskan LUX multimode reader.

**In vivo Biocompatibility Evaluation**

Normal C57BL/6 mice received topical instillation of PBS, SR, Sta@SR, or Sta@MSR at the same concentrations used for therapeutic experiments. Eye drops were administered per eye three times daily for 30 days. Body weight was recorded every 3 days. Intraocular pressure was measured using a rebound tonometer. Corneal and conjunctival conditions were assessed by slit-lamp biomicroscopy. Sodium fluorescein staining was performed to evaluate corneal epithelial integrity. Fundus photography and anterior-segment OCT imaging were performed using an ophthalmic imaging system. Corneal immunofluorescence staining for TNF-α and IL-6 was performed on cryosections as described above. At the end of the observation period, mice were euthanized, and major organs, including the heart, liver, spleen, lung, and kidney, were harvested, fixed in 4% paraformaldehyde overnight, paraffin-embedded, sectioned at 5 μm, and stained with H&E for histopathological evaluation.

**Supplementary Figures**

**Figure S1.** Morphological characterization of dendritic mesoporous silica nanoparticles (DMSNs) and ruthenium-modified dendritic mesoporous silica nanoparticles (Silica-Ruthenium, SR). a) TEM image of DMSNs. b) TEM image of SR. Scale bar: 100 nm.

**Figure S2.** SDS-PAGE protein profiles of whole macrophage lysates, isolated macrophage membranes, and Sta@MSR, showing the preservation of macrophage membrane-associated protein patterns after membrane cloaking.

**Figure S3.** Concentration-dependent DPPH radical scavenging ability of Sta@MSR. a) UV–vis absorption spectra of DPPH after incubation with Sta@MSR at different concentrations. b) EPR spectra of DPPH radicals after treatment with Sta@MSR at different concentrations.

**Figure S4.** Time- and concentration-dependent cellular uptake of AF488@MSR in HCECs. a) Representative confocal fluorescence images showing the cellular uptake of AF488@MSR in HCECs. Nuclei were stained with DAPI. Scale bars, 50 μm. b) Quantitative fluorescence intensity analysis of AF488@MSR uptake under different incubation times and concentrations (n = 3). Data are presented as mean ± SEM. Statistical significance was determined by one-way ANOVA with appropriate multiple-comparison tests. ns, not significant, ***p* < 0.01, ****p* < 0.001, *****p* < 0.0001.

**Figure S5.** Enhanced cellular uptake of AF488@MSR in LPS-stimulated HCECs. a) Representative images showing the cellular uptake of AF488@MSR in PBS-treated and LPS-stimulated HCECs. AF488@MSR is shown in green, F-actin stained with phalloidin, and nuclei stained with DAPI. Scale bars, 15 or 5 μm. b) Quantitative analysis of AF488 fluorescence intensity in HCECs under different conditions (n = 3). Data are presented as mean ± SEM. ***p* < 0.01.

**Figure S6.** Concentration- and time-dependent attenuation of intracellular oxidative stress by Sta@MSR in HCECs. a) Representative CellROX fluorescence images showing intracellular oxidative stress-associated fluorescence in H₂O₂-stimulated HCECs following treatment with different concentrations of Sta@MSR or for different durations. Scale bars, 50 μm. b) Quantitative analysis of CellROX fluorescence intensity under different treatment concentrations and durations (n = 3). Data are presented as mean ± SEM. Statistical significance was determined by one-way ANOVA with appropriate multiple-comparison tests. ns, not significant; **p* < 0.05, ***p* < 0.01, ****p* < 0.001, *****p* < 0.0001.

**Figure S7.** Inhibitory effects of Sta@MSR on HUVEC tube formation. a) Representative images of HUVEC tube formation after treatment with PBS, free Stattic, SR, Sta@SR, or Sta@MSR. Scale bars, 100 μm. b-d) Quantitative analysis of total tube length, number of meshes, and number of junctions, respectively (n = 3). Data are presented as mean ± SEM. Statistical significance was determined by one-way ANOVA followed by Tukey’s post hoc multiple-comparison test. **p* < 0.05, ***p* < 0.01, and *****p* < 0.0001.

**Figure S8.** Heatmap of differentially expressed genes among normal, PBS-treated, and Sta@MSR-treated corneas. The heatmap shows the overall expression patterns of differentially expressed genes across the three groups. PBS-treated corneas exhibited a distinct injury-associated transcriptional profile compared with normal corneas, while Sta@MSR-treated corneas showed a partially restored expression pattern toward the normal state.

**Figure S9.** Gene Ontology (GO) enrichment analysis of differentially expressed genes between PBS- and Sta@MSR-treated corneas. GO enrichment analysis revealed that Sta@MSR-regulated genes were enriched in biological processes related to inflammatory signaling, immune-cell migration, extracellular matrix organization, and angiogenesis-associated responses.

**Figure S10.** HCECs and HCSCs were treated with Sta@MSR for 24 h, and cell viability was determined by CCK-8 assay (n = 3). Data are presented as mean ± SEM.

**Figure S11.** Effect of Sta@MSR on HCEC wound closure. a) Representative images of scratch wound closure in HCECs treated with PBS or Sta@MSR at 0, 12, and 24 h. Blue outlines indicate the wound boundaries identified during image analysis. b) Quantitative analysis of relative wound closure at 12 and 24 h, normalized to the initial wound area at 0 h (n = 3). Data are presented as mean ± SEM. ns, not significant.

**Figure S12.** The full gels of Western blot data for Figure 1b.

**Figure S13.** The full gels of Western blot data for Figure 2i.

**Figure S14.** The full gels of Western blot data for Figure 7k.
